# A Scoping Review of Interventions Designed to Support Parents With Mental Illness That Would Be Appropriate for Parents With Psychosis

**DOI:** 10.3389/fpsyt.2021.787166

**Published:** 2022-01-27

**Authors:** Jessica Radley, Nithura Sivarajah, Bettina Moltrecht, Marie-Louise Klampe, Felicity Hudson, Rachel Delahay, Jane Barlow, Louise C. Johns

**Affiliations:** ^1^Department of Psychiatry, University of Oxford, Oxford, United Kingdom; ^2^Oxford Health NHS Foundation Trust, Oxford, United Kingdom; ^3^Department of Experimental Psychology, University of Oxford, Oxford, United Kingdom; ^4^School of Psychological Science, Faculty of Life Sciences, University of Bristol, Bristol, United Kingdom; ^5^Department of Social Policy and Intervention, University of Oxford, Oxford, United Kingdom

**Keywords:** review, intervention, psychosis, parenting, children, mental health

## Abstract

The experience of psychosis can present additional difficulties for parents, over and above the normal challenges of parenting. Although there is evidence about parenting interventions specifically targeted at parents with affective disorders, anxiety, and borderline personality disorder, there is currently limited evidence for parents with psychotic disorders. It is not yet known what, if any, interventions exist for this population, or what kinds of evaluations have been conducted. To address this, we conducted a scoping review to determine (1) what parenting interventions have been developed for parents with psychosis (either specifically for, or accessible by, this client group), (2) what components these interventions contain, and (3) what kinds of evaluations have been conducted. The eligibility criteria were broad; we included any report of an intervention for parents with a mental health diagnosis, in which parents with psychosis were eligible to take part, that had been published within the last 20 years. Two reviewers screened reports and extracted the data from the included reports. Thirty-eight studies of 34 interventions were included. The findings show that most interventions have been designed either for parents with any mental illness or parents with severe mental illness, and only two interventions were trialed with a group of parents with psychosis. After noting clusters of intervention components, five groups were formed focused on: (1) talking about parental mental illness, (2) improving parenting skills, (3) long-term tailored support for the whole family, (4) groups for parents with mental illness, and (5) family therapy. Twenty-three quantitative evaluations and 13 qualitative evaluations had been conducted but only eight interventions have or are being evaluated using a randomized controlled trial (RCT). More RCTs of these interventions are needed, in addition to further analysis of the components that are the most effective in changing outcomes for both the parent and their children, in order to support parents with psychosis and their families.

## Introduction

Parenting can be challenging for parents who experience psychosis. Psychotic symptoms include positive symptoms, such as hallucinations and delusions, and negative symptoms, such as apathy and blunted affect (1). Psychosis has other associated difficulties, including memory and concentration problems, co-morbid affective conditions, difficulties in understanding the mental states of others, and sensitivity to stress and poor sleep (2). Individuals who experience psychosis also often have to cope with side-effects from anti-psychotic medication, particularly sedation (3). These symptoms and side-effects can make it more difficult for parents to empathize with their children and communicate clearly, and to offer the consistent, responsive care required for healthy child development (4–6). A diagnosis of psychosis is also associated with adverse childhood experiences, such as sexual, physical, and emotional abuse (7, 8), which may affect parents forming stable attachments with their own children (9). During an acute episode of psychosis, parents may find it difficult to care for their children at all (10) and family life can be disrupted if the parent is hospitalized (11).

Although not all parents with psychosis experience problems with their parenting, those who report more severe symptoms and a longer duration of illness are more likely to show such problems (12). However, it is not only symptom severity that makes parenting challenging; a diagnosis of psychosis is associated with many environmental factors that can precipitate further difficulties, including being a single parent, (13), poor social support (14), financial instability (15, 16), and unemployment (17). These socioeconomic factors, in turn, are associated with more frequent experiences of psychiatric symptoms (18), and predict a poorer quality of parenting (14). This social adversity may even be more detrimental to parenting than the direct effects of parental mental illness (19).

Intervening with these families could lead to positive outcomes for both the parent and their child. Elements of a successful intervention may include crisis management in anticipation of future relapses (20), links to other services to provide parents with practical support (21), as well as help with parenting skills (22). Custody loss is experienced by parents with serious mental illness more often than parents without mental health problems (23, 24). It is a fear of many of these parents (25), which can mean some parents are reluctant to seek help and take part in parenting interventions (26). Therefore, appropriate interventions should acknowledge the parenting role as an important part of recovery (27, 28), which could then help to prevent custody loss (29), while also reducing the risk of the children developing mental health problems themselves (30). Research with children of parents with mental illness has shown that they want to understand their parent's mental illness (21), and explanation about their parent's illness may be protective for these children (31).

Parenting interventions aim to improve parenting skills and relationships within the family (32) by providing parents with skills focused on encouraging positive behavior and education about child development (33, 34). Parenting interventions often have a focus on parents whose children are demonstrating behavioral difficulties (35) and there is good evidence that they can reduce emotional and behavioral difficulties for these children (34). More recently some of these interventions have been amended to support parents with mental health problems [e.g., (36)] or the intervention has been used in its original form with a group of parents with a mental health diagnosis, like Triple P (37) and Tuning into Kids (38). Parenting interventions that are tailored toward parents with mental health difficulties were initially designed for parents with affective disorders (39), and this client group is still the focus of many such programs (40, 41). Specific programs have, however, also been developed for parents with other types of mental health diagnoses, such as anxiety (42), and personality disorders (43). However, the availability of interventions for parents with psychosis is limited, with the majority focusing on mothers experiencing postpartum psychosis (44), leaving a significant gap with regard to interventions for parents with psychosis who have older children. To address this gap, we need to know which interventions exist, as well as what elements these interventions contain in order to address the needs of families with parental psychosis. Ways in which these needs may be addressed include planning for periods of hospitalization (20) and improving parents' ability to understand their child's mental states (45).

This review is the sequel to a Cochrane systematic review (46) in which a search was undertaken to identify the evidence for parenting interventions designed to improve parenting skills or the parent-child relationship in parents with psychosis. However, only one study was identified, which was published almost 40 years ago. Other similar reviews include Schrank et al. (47) and Suarez et al. (48). Schrank et al. (47) conducted a systematic review of interventions that reported quantitative findings, in which at least 50% of the participants were parents with severe mental illness (which they defined as psychotic or bipolar disorders) and identified 15 interventions. Suarez et al. (48) conducted a scoping review for interventions for mothers with any kind of mental illness that had described some kind of outcome for the study participants, and identified nine interventions.

The aim of this review is to identify what interventions are available for parents with psychosis, to describe the content of these interventions, and provide a narrative synthesis about existing evaluations and what they have found.

### Research Questions

1. What parenting interventions have been developed for parents with psychosis (either specifically for, or accessible by, this client group)?
2. What are the components of these interventions?
3. What kinds of evaluations have been conducted to determine their acceptability and effectiveness, and what do the findings show?

## Methods

The current scoping review systematically searched all relevant databases, trial registries and gray literature with the aim of mapping current research about parenting interventions for parents with psychosis. In contrast to Radley et al. (46), Schrank et al. (47), and Suarez et al. (48), it treated as eligible any report of an intervention regardless of the level of evaluation to which it has been subjected. The inclusion criteria were also broader in that any intervention for parents with mental health problems was included. Interventions for parents with specific mental health diagnoses in which parents with psychosis were not eligible to take part were excluded from this review since these interventions may not be appropriately designed to address the needs of parents with psychosis. In order to address the gap that exists around interventions for parents with psychosis with older children, we only included studies in which the children were older than 2 years. This review was also limited to papers published within the last 20 years in order to describe what may be currently available for these parents.

This manuscript is written in accordance with the PRISMA guidance for reporting scoping reviews (49).

### Protocol and Registration

The protocol was uploaded to the Open Science Framework (https://osf.io/3d7t9/) in May 2021.

### Eligibility Criteria

This review followed the scoping review framework by Arksey and O'Malley (50). It included peer-reviewed papers, trial registries, and gray literature including Ph.D. theses, websites, and preprints. To be included, reports had to be written in the last 20 years and include an evaluation or description of an intervention for parents with a mental health diagnosis, in which parents with psychosis were eligible to take part. The intervention could be child-focused, parent-focused, or family-focused as long as there was a specific component for the parent.

The following were excluded:

- Reviews.
- Interventions designed for the children of parents with a mental health diagnosis with no parenting component.
- Interventions designed to improve service-response or healthcare professional knowledge of parental mental illness with no parenting component.
- Interventions that excluded parents with psychosis.
- Interventions that targeted parents with children under the age of 2 years.

Records were also excluded if they were written in any language apart from English. However, it became clear that a large number of potentially eligible German papers were being excluded. It was decided the review would be incomplete without consideration of these papers, and therefore a German-speaking author, BM, reviewed all of these records at full-text stage.

### Information Sources

Eight databases were searched on January 11^th^ 2021, and updated on November 6^th^ 2021, for records published since January 2001 in PsycINFO, Embase, MEDLINE, CINAHL, ASSIA, Scopus, Web of Science, and ProQuest Dissertations and Theses. The search strategy was designed in collaboration with an experienced librarian and altered to suit the requirements of each database. The records found in each database were deduplicated after importing them into EndNote. The ICTRP was searched for trial registries.

Once the included reports had been identified, their reference lists were searched for further eligible reports. Finally, titles of included reports were entered into Google Scholar to find more recent published work that had cited these reports. This was done in April 2021, and updated in November 2021.

JR searched the reference lists of any similar reviews known to the authors or any reviews found during the search for any additional eligible reports in April 2021.

### Search Strategy

An original search strategy was created in collaboration with a librarian. After trialing this, it was clear that more general words for “mental health” needed to be added to retrieve papers in which parents with psychosis might have been involved, but where psychosis was not mentioned in the title or abstract. It also became clear that searches using index subject headings were not as effective as searches using key terms. Therefore, only searches using key terms were used for the final search strategy. The full electronic search strategy for MEDLINE was as following:

1. ((schizophreni^*^ or smi or “serious mental illness” or “severe mental illness” or psychosis or paranoi^*^ or “mental health” or “mental^*^ ill^*^” or “mental^*^ disorder^*^” or “mental^*^ impair^*^” or “psychiatric”) adj4 (parent^*^ or mother^*^ or father^*^ or maternal^*^ or paternal^*^)).ab,ti.
2. (psychotherap^*^ or therap^*^ or intervention^*^ or train^*^ or education^*^ or program^*^).ab,ti.
3. limit 1 to yr = “2001-Current”
4. limit 2 to yr = “2001-Current”
5. 3 and 4

A similar search strategy was adapted for other databases, trial registries, preprint servers and websites. Websites were searched using Google Advanced, by limiting the domain to org.uk, gov, gov.uk, com.au, nhs.uk, or org.

### Selection of Sources of Evidence

After the records obtained from the database search were deduplicated using EndNote, they were imported onto Rayyan, which is an online platform designed for multiple reviewers to work on systematic reviews (51). Reviewers are kept blind to each other's decisions, and are able to mark records as “include,” “exclude,” or “maybe” and can also mark exclusion reasons or add notes. This process was used to determine which records would be brought forward to full text review. All records were reviewed by JR, then FH and MLK each screened 50% of records, such that each record was screened twice. Every record that was deemed to be eligible by at least one researcher was brought forward to full text review (i.e., if there was a disagreement, this record was brought forward to full text review).

**Figure 1:**
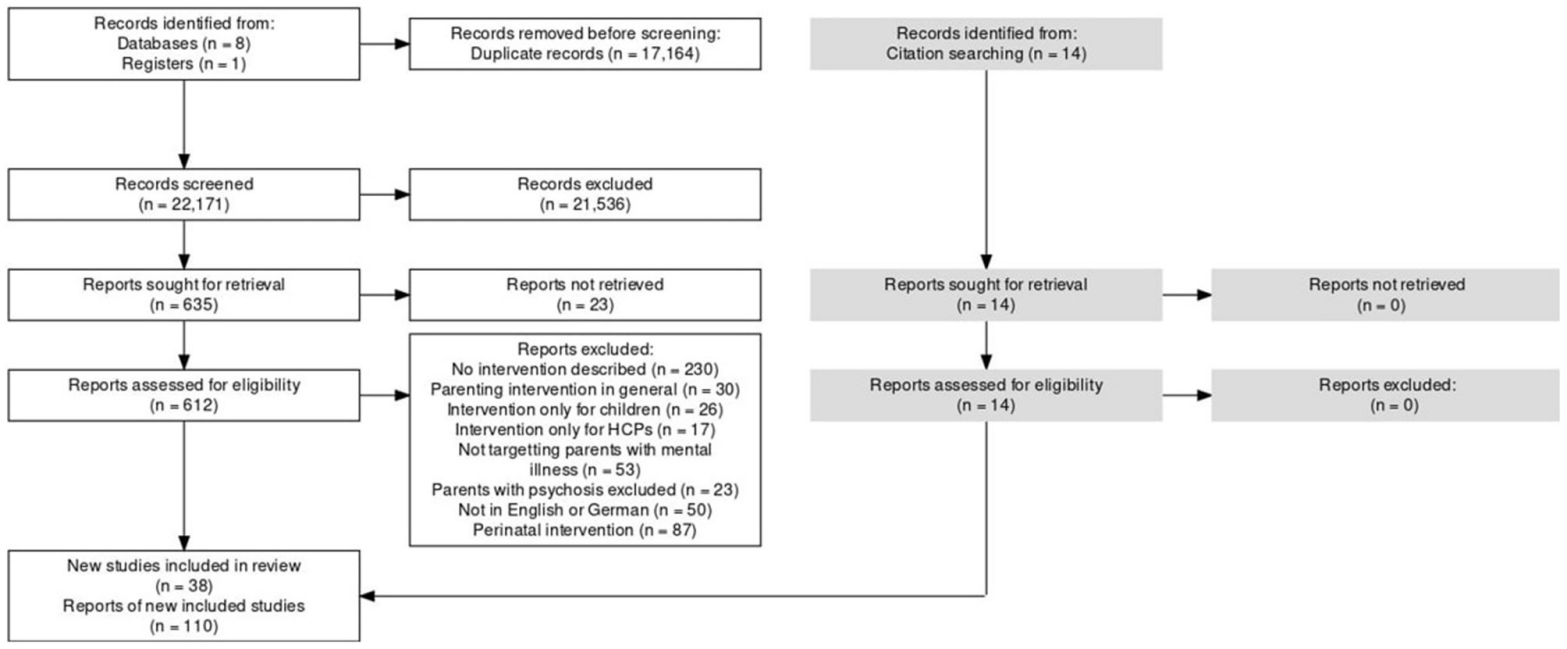
Flow diagram of identification of reports.

Full text review was completed using Excel. JR retrieved the full texts for every paper. NS reviewed a random sample of 25% of the records, and a Cohen's kappa of 0.90 was achieved (52). The German records were screened at full-text stage by BM only. Reasons for exclusion are detailed in Figure 1.

Trial registries, preprint servers and websites were reviewed by JR only.

### Data Charting Process

JR, LJ, and JB discussed the included papers and decided which details to extract from each report in order to satisfy the research questions. An excel form was created to capture this data with limits in terms of what values could be entered under each section. JR extracted data from all reports, then NS and RD extracted data from 50% of the papers each, such that each included paper underwent double data extraction. Where information was not available in the paper, the relevant field in the data extraction form was left blank. Disagreements were resolved through discussion. BM extracted data from the included reports which were written in German.

### Data Items

Each data item was a study of an intervention. Data were extracted from each report on (1) location of the intervention, (2) who the intervention was intended for, (3) who delivers the intervention and how much training they receive, and (4) the format of the intervention. When an intervention provided separate components for the parent and the child, only components relevant to the parent intervention were reported.

Details about the components of each intervention were extracted e.g., explaining mental illness to children, psychoeducation, parenting skills, case management. Where the same intervention had been trialed by different teams but no adjustments had been made to the components, it was collapsed into one item.

If an evaluation had been completed, or registered as a protocol, participants' demographic details, and the design and results of the evaluation were extracted.

Qualitative evaluations were only included when participants were given the opportunity to answer open-ended questions, as part of a survey or an interview. When available, the themes produced from a qualitative analysis were extracted, otherwise the most salient elements from the qualitative research were extracted. If multiple intervention members were interviewed (e.g., parent, child, facilitator), only the data produced by the parents that were specific to the parenting intervention were extracted.

For quantitative evaluations, outcome measures related to the parent or child were extracted, and classified into “child behavior,” “child psychosocial,” “child quality of life,” “parenting,” “parent psychosocial,” or “parent quality of life,” and any significant differences obtained on these measures were indicated.

The final data charting form can be found in Supplementary File 1.

### Synthesis of Results

Once the data charting form was completed, frequency data on the interventions was reported. After charting the components of each intervention, interventions with similar components were grouped into five categories. After inspection of the clusters of components in these similar interventions, these categories were named: (1) Talking about parental mental illness, (2) Improving parenting skills, (3) Long-term tailored support for the whole family, (4) Groups for parents with mental illness, and (5) Family therapy. A narrative summary was provided for the qualitative and quantitative evaluations of interventions.

## Results

### Selection of Records

After duplicates were removed, a total of 22,171 records were screened by at least two reviewers at the title and abstract stage. If at least one reviewer decided a record should be included to full text stage, it was brought forward, which was the case for 635 records. Of these, 23 could not be retrieved from library journal databases, and the remainder were assessed for eligibility at full text stage. The main reason for exclusion (*n* = 502) was that the report did not describe an intervention (see Figure 1 for further detail). A total of 96 reports were included in the review. After looking at their reference lists as well as using Google scholar to search for more recent reports that had cited them, 14 more reports were found, making a total of 110. Most interventions had multiple reports describing them, such that the 110 reports described 38 studies of interventions, which accounted for 34 interventions in total. Three reports were written in German. All reports that were included can be found in Supplementary File 2.

Records were identified from database searches and trial registries. No additional records were identified through organizational websites, preprint servers or through searching the reference lists of other similar reviews.

### Characteristics of Interventions

Many interventions had been delivered in more than one country. The country that had developed the most interventions was Australia (*n* = 7), followed by the UK (*n* = 6), Germany (*n* = 5), the Netherlands (*n* = 5), and the USA (*n* = 5). There was also a report of an intervention from each of the Scandinavian countries: Sweden (*n* = 3), Finland (*n* = 2), Denmark (*n* = 1), and Norway (*n* = 1). Switzerland and Israel had two interventions each and Portugal and Ireland had one each. Table 1 presents the data extracted from each of the included studies.

**Table 1:** Characteristics of 38 studies of interventions.

**Intervention and authors of primary report(s)**	**Country**	**Parent diagnosis and child age**	**Who takes part in intervention**	**Who delivers intervention and training**	**Setting of intervention**	**Referral method**	**Group or Individual**	**Length of intervention**	**Manualized**
BROSH program (53)	Israel	MI, 0–18	Whole family	Mental health professional or social worker	Home	Adult mental health or child services	Individual	3 h weekly meeting for 2 years	No
Child and family inclusive programme (54, 55)	Australia	MI, 4–18	Whole family	Mental health professional or social worker	Community or home	Adult mental health or self-referral	Individual	3–8 60–90 min sessions	No
Child resilience programme (56)	USA (Indiana)	SMI, 8–18	Whole family	*Unknown*	Community	Adult mental health	Both	7–8 weekly individual family sessions 2+ monthly group therapy	No
Child Talks+ (57, 58)	Norway, Portugal, the Netherlands	MI, 0–18	Whole family	Mental health professional or social worker Two days	Community or home	Adult mental health	Individual	Four weekly or biweekly 1 h sessions	Yes
CHIMPS intervention (59)	Germany and Switzerland	MI, 3–19	Whole family	Mental health professional Two days	Community	Adult mental health	Individual	8 × 60–90 min sessions over a period of 6 months	Yes
Counseling and support service (60)	Germany	MI	Whole family	Mental health professional	Community	Adult mental health	Individual		No
Effective child and family program (61, 62)	Finland	MI	Whole family	Mental health professional or social worker Seventeen days	Community or home	Adult mental health	Individual	6–8 sessions for Family Talk OR 2–3 for Let's Talk 1 family meeting	Yes
Family options (63, 64)	USA (Massachusetts)	SMI, 18 months−16	Whole family	Psychology graduates	Home	Adult mental health or child services	Individual	Weekly meetings for 12 months	No
Family Talk (65)	Germany	MI	Whole family	*Unknown*	Community	Self-referral	Both	2 × 90 min group sessions for parents 5 group sessions for children One individual family session Over 3 months	Yes
Family Talk (66)	Ireland	MI, 5–18	Whole family	Mental health professional or social worker Online training–15 h and monthly supervision	Community or home	Adult mental health	Individual	7 weekly 60–90 min sessions	Yes
Family Talk (67)	Sweden	MI, 8–18	Whole family	Mental health professional or social worker 5 days of theory, 5 days of supervision in a year	*Unknown*	Adult mental health	Individual	6 or 7 sessions	Yes
Family Talk (68, 69)	Sweden	Psychosis, 8–17	Whole family	Mental health professional or social worker	*Unknown*	*Unknown*	Individual	6 or 7 sessions	Yes
FWA Newpin service (70)	UK (London)	MI, 0–5	Parent and child	Social worker	Community	*Unknown*	Both	Meetings held twice a week	No
Godparents programme (71)	Switzerland	MI, 0–18	Whole family	Non-professionals Introductory event, regular peer supervision, two-four supervisions with coordinator a year	Home	Adult mental health or child services	Individual	Regular meetings for at least 3 years	No
Integrated family treatment (72)	USA (New Hampshire)	SMI	Parent and child	Mental health professional	Home	Adult mental health	Individual	1–5 years of sessions	No
Invisible children's project (20)	USA (New York)	MI	*Unknown*	Social worker	*Unknown*	Child services referral	*Unknown*	*Unknown*	No
KidsTime (73, 74)	UK, Germany, Spain	MI	Parent and child	Mental health professional or social worker Two days	Community	Adult mental health or child services or self-referral	Group	Monthly meetings lasting 2.5 h	Yes
KopOpOuders (22)	The Netherlands	MI, 1–21	Parent	Mental health professional	Online	Adult mental health or child services or self-referral	Group	8 weekly 90 min sessions	Yes
Let's talk about children (75, 76), ACTRN12616000460404	Finland, Sweden, Australia	MI, 0–18	Parent	Mental health professional Two days online and 4 h face to face	*Unknown*	Adult mental health	Individual	2 or 3 weekly 60 min sessions	Yes
Let's talk about children booklet (77)	Australia	MI, 0–18	Parent	Self-help	Community or home	Adult mental health	Individual	Open-ended	No
Living with under fives (78, 79)	Australia	SMI, 0–5	Parent and child	Occupational therapist	Community	Adult mental health or child services	Group	Weekly meetings lasting 2 h	No
Parenting internet intervention (80)	USA (Pennsylvania)	SMI, 0–18	Parent	Self-help	Online	Self-referral	Individual	12 weekly 30 min sessions	Yes
Parenting with success and satisfaction workbooks (81–83)	The Netherlands	SMI, 0–21	Parent	Self-help with option of Mental health professional Four days	Community or home	Adult mental health	Both	Weekly meetings for a year	Yes
Preventive basic care management (PBCM) (84, 85)	The Netherlands	MI, 3–10	Whole family	*Unknown*	Home	Adult mental health	Individual	18 months	No
SEEK (86)^*^	Germany	SMI	Parent and child	Mental health professional	Child inpatient unit	Adult mental health or child services	Group	6 × 90 min sessions over 5 weeks	Yes
Strengths based parenting programme (87)	Australia	MI	Parent	Mental health professional	Community	Adult mental health or self-referral	Group	5 weekly 2 h sessions	No
The lighthouse (leuchtturm) parenting programme (88)^*^	Germany	SMI, 0–14	Parent	Psychologist, social worker, psychiatrist, nurses	Adult inpatient unit	Adult mental health or self-referral	Both	5 individual sessions (2 with video feedback) One session with care worker 4 group sessions Weekly over 12 weeks	Yes
Therapeutic group (89)	Israel	MI	Parent	Mental health professional or social worker	Community	Adult mental health or child services	Group	Weekly meetings for 21 months	No
Think family whole family programme (90)	UK (Leicester)	MI	Whole family	Mental health professional or social worker Two days	*Unknown*	*Unknown*	Individual	8 sessions	Yes
Triple P (91)^*^	Germany	SMI, 2–10	Parent	Mental health professional 10 sessions of training	Community	Adult mental health or child services	Individual	8–10 weekly 50–60 min sessions	Yes
Triple P – every parent's self-help workbook (92)	UK (Manchester)	MI, 2–12	Parent	Self-help with option of mental health professional 45–60 min	Home	Child services referral	Individual	Booklet is completed over 10 weeks	Yes
Triple P – every parent's self-help workbook (93)	UK (Manchester)	Psychosis, 3–10	Parent	Self-help with option of mental health professional	Home	Adult mental health or child services	Individual	10–14 weekly visits for 1.5 h	Yes
Triple P + CBT (37)	Germany	MI, 1.5–16	Whole family	Mental health professional	Community	Adult mental health	Both	25–45 sessions CBT 8–10 sessions Triple P Weekly or bi-weekly sessions for 6–12 months	Yes
Triple P + mental health components (36)	Australia	MI, 2–12	Parent	Mental health professional or social worker	Community or home	Adult mental health or child services or self-referral	Both	6 weekly 2.5–3 h group + four individual visits	Yes
Tuning into kids (38)	Australia	MI, 3–12	Parent	Mental health professional	Community	Adult mental health	Group	6 weekly 2 h sessions	Yes
VIA family (94)	Denmark	SMI, 6–12	Whole family	Child psychiatrist, child psychologist, adult mental health nurse social worker, and a family counselor	Community or home	Adult mental health	Individual	1–2 sessions introduction 2–4 sessions lifeline and history 6–8 sessions psychoeducation 3–10 sessions Triple P 8 sessions groups for children and parents All over 18 months	No
You are okay (95, 96)	The Netherlands	MI, 10–20 with mild individual disability	Parent and child	Self-help with option of support from social worker	Online	Child services referral	Individual	5 sessions online for parents + 10 weekly support group sessions for children	Yes
Young SMILES (97, 98)	UK (Manchester)	SMI, 6–16	Whole family	Mental health professional or social worker Three days	Community	Adult mental health or child services	Group	5 weekly 2 h sessions	Yes

*MI, mental illness; SMI, severe mental illness*.

* *Indicates paper written in German*.

Most interventions were designed either for parents with any mental illness or parents with severe mental illness, as defined by the study authors. Only two interventions were trialed with a group of parents with psychosis—*Triple P* (93) and *Family Talk* (68)—neither of which had been adapted from their original format. Eighteen interventions were designed for the whole family, six were for the affected parent and their child(ren) and 13 were for the affected parent only. Most interventions were led by a mental health professional or a social worker, or were in the form of self-help except for *Family Options* which is led by a graduate in psychology (63) and the *Godparents programme* which is led by a non-professional (71).

Many interventions were designed to be delivered in an outpatient community setting (*n* = 13), seven in a home setting, and eight interventions either in a community and home setting, or involved both a community and a home element. Three interventions were provided online, and it wasn't possible to determine the location of five interventions. Most interventions were delivered on a one-to-one basis (*n* = 22), a smaller number having been designed to be delivered using a group format (*n* = 8), or using both individual and group components (*n* = 7). Group interventions were more likely to be for the parent only or for both the parent and the child with a parent group and a child group being held separately.

The shortest intervention was *Let's Talk about Children* in either the meeting format, with two to three sessions (75), or via a self-help booklet (77). Some interventions were open-ended, meaning the parents could attend for as long as they liked [e.g., (67)] and the *Godparents programme* lasted for at least 3 years (71).

### Intervention Components

Out of the 38 studies included in this review, four described *Beardslee's Family Talk* (65–68) and two described the *Triple P self-help workbook* (92, 93). Therefore, these 38 studies described 34 unique interventions. Of the 34 interventions listed in Table 2, most covered parenting skills (*n* = 21), aimed to strengthen the parent–child relationship (*n* = 18) or contained psychoeducation on child development (*n* = 17). Many interventions also had a focus on the child by including psychoeducation for the parent either on how their illness might impact upon their child (*n* = 16) or explaining mental illness to the child (*n* = 16). The intervention that comprised the most components was *VIA Family*, which contained 12 out of the 20 total components. Interventions were grouped into the following five categories depending on their focus.

**Table 2:** Components of 34 interventions, separated into five categories.

**Intervention and Primary report(s)**	**Explaining mental illness to child(ren)**	**Psycho-education on how PMI impacts on child**	**Psycho-education on mental health**	**Psycho-education on child develop-ment**	**Chance for family to talk about experiences of PMI**	**Parent-child relationship**	**Parenting skills**	**Parent well-being or self-care**	**Parent social support**	**Parent emotional support**	**Peer support**	**Money manage-ment**	**Goal setting**	**Crisis planning for periods of poor MH**	**Family therapy**	**Case manage-ment**	**Interagency or multi team collaboration**	**Signposting to other supportive agencies**	**Mentalizing component**	**Separate child element**
**TALKING ABOUT PARENTAL MENTAL ILLNESS**																				
Family Talk (65–69)	**X**	**X**	**X**		**X**															**X**
Let's Talk about children (75, 76), ACTRN12616000460404	**X**	**X**			**X**															
Let's Talk about Children booklet (77)	**X**	**X**			**X**															
Effective Child and Family Program (61, 62)	**X**	**X**			**X**												**X**	**X**		
CHIMPS intervention (59)	**X**	**X**	**X**		**X**	**X**		**X**	**X**						**X**					
Child Talks+ (57, 58)	**X**	**X**	**X**		**X**		**X**			**X**										
Child and family inclusive programme (54, 55)	**X**				**X**															
KidsTime (73, 74)	**X**	**X**	**X**		**X**				**X**		**X**						**X**	**X**		**X**
**IMPROVING PARENTING SKILLS**																				
Triple P self-help workbook (92, 93)				**X**		**X**	**X**						**X**							
Triple P + CBT (37)							**X**													
Triple P + mental health components (36)		**X**		**X**		**X**	**X**							**X**						
Triple P (91)*	**X**	**X**	**X**	**X**		**X**	**X**	**X**					**X**	**X**						
Tuning into kids (38)				**X**		**X**	**X**			**X**	**X**								**X**	
The lighthouse (leuchtturm) parenting programme (88)*				**X**		**X**	**X**		**X**				**X**				**X**	**X**	**X**	
Strengths based parenting programme (87)	**X**	**X**	**X**	**X**		**X**	**X**	**X**	**X**					**X**				**X**		
KopOpOuders (22)				**X**		**X**	**X**		**X**					**X**						
You are okay (95, 96)	**X**	**X**					**X**		**X**											**X**
Parenting internet intervention (80)		**X**		**X**		**X**	**X**	**X**			**X**									
Parenting with success and satisfaction workbooks (81–83)							**X**	**X**					**X**					**X**		
**LONG-TERM TAILORED SUPPORT FOR THE WHOLE FAMILY**																				
Invisible children's project (20)							**X**		**X**	**X**				**X**		**X**	**X**	**X**		
Family options (63, 64)										**X**	**X**	**X**	**X**	**X**		**X**	**X**	**X**		
Integrated family treatment (72)				**X**		**X**	**X**		**X**	**X**							**X**	**X**		
VIA family (94)		**X**	**X**	**X**		**X**	**X**		**X**			**X**		**X**		**X**	**X**	**X**		**X**
Preventive basic care management (PBCM) (84, 85)				**X**					**X**							**X**	**X**	**X**		**X**
Godparents programme (71)					**X**				**X**	**X**										**X**
BROSH program (53)	**X**			**X**		**X**	**X**	**X**				**X**			**X**	**X**	**X**		**X**	**X**
**GROUPS FOR PARENTS WITH MENTAL ILLNESS**																				
Living with under fives (78, 79)				**X**		**X**	**X**		**X**					**X**			**X**	**X**		**X**
FWA newpin service (70)						**X**		**X**	**X**		**X**								**X**	
Therapeutic group (89)	**X**			**X**		**X**	**X**			**X**	**X**						**X**	**X**		
Young SMILES (97, 98)	**X**			**X**		**X**	**X**													**X**
SEEK (86)*	**X**		**X**	**X**	**X**	**X**	**X**	**X**		**X**				**X**			**X**	**X**		**X**
**FAMILY THERAPY**																				
Child resilience programme (56)	**X**	**X**	**X**		**X**	**X**	**X**		**X**				**X**	**X**	**X**					**X**
Think family whole family programme (90)		**X**	**X**		**X**								**X**	**X**	**X**			**X**		
Counseling and support service (60)		**X**		**X**											**X**	**X**				
Total	**16**	**16**	**10**	**17**	**12**	**18**	**21**	**8**	**14**	**8**	**6**	**3**	**7**	**11**	**5**	**6**	**12**	**14**	**4**	**11**

*PMI, parental mental illness*.

#### Talking About Parental Mental Illness

Eight interventions focused on explaining parental mental illness to the child[ren] in the family and giving family members the space to talk about their experiences of parental mental illness. *Family Talk* was originally designed in the USA to target families with affective disorders (39) and has since been used with parents with any mental illness. Depending on its adaptation, it usually involves six to eight sessions, includes separate meetings for the parents and the children, and concludes with whole family meetings. *Let's Talk about Children* is a similar, but much shorter intervention in which the children are not invited to the meetings, and instead the parents are given advice on how to talk about their mental illness to their child (76). *Let's Talk about Children* also exists in a booklet form (77). The *Effective Child and Family Program* (61) offers either *Family Talk* or *Let's Talk about Children*, as well as self-help material with the potential for a multiagency meeting for the family, if any problems are identified. The *CHIMPS intervention* in Germany (59) has adapted *Family Talk* by including psychodynamic elements. *Child Talks*+ (57) aims to enable the parents to explain mental illness to their children and for family members to get a chance to talk about their experiences. It consists of four meetings, with the first two being only with the parents, and the children attending the final two. The *Child and Family Inclusive Program* (54) has a similar focus but allows families to choose whether children are seen together with the parents, or separately. *KidsTime* (73) is an intervention that both children and parents attend, in which children take part in a drama group and parents take part in a parent group. Everyone meets at the end of the session to watch the children perform, and the content of these performance often centers on the parent's mental illness.

#### Improving Parenting Skills

Eleven of the interventions had a focus on improving parenting skills. Four interventions (36, 37, 91, 93) were based on the *Triple P*, originally designed for the parents of children with behavioral difficulties (99). *Triple P* teaches parents about enhancing their relationship with their children, encouraging certain behaviors, discouraging others, and setting clear boundaries (99). In this review, the *Triple P Every Parents' Self-Help Workbook* (92, 93) was used for parents with mental illness, and Stracke et al. (37) combined *Triple P* with cognitive behavioral therapy. Both Phelan et al. (100) and Kuschel et al. (91) add two additional components about parental mental health to the *Triple P* syllabus. Two interventions were based on mentalization. The *Lighthouse (Leuchtturm) Parenting Programme* (88) is rooted in mentalization-based therapy, and aids parents in better understanding their child's mental states, and teaches behavioral management skills. *Tuning into Kids* focuses on teaching parents how to recognize and respond to their child's emotions (101), and Isobel et al. (38) trialed it with parents with mental illness. McFarland et al.'s (87) strengths based parenting programme took elements from *Triple P* and *Tuning into Kids*, and also had a focus on talking about parental mental illness to the child. *KopOpOuders* (22) is an online course which covers boundary setting, communicating, child development and emergency planning. *You are Okay* (95) is an intervention for parents with mental illness whose children have an intellectual disability. It has a support group for the children as well as an online course for parents which is based on the content of *KopOpOuders*. The *Parenting Internet Intervention* designed by Kaplan et al. (80) contained modules on child development, stress management, the effects of parental mental illness, and setting boundaries. *Parenting with Success and Satisfaction (PARSS)* (81) is a series of three workbooks, and has a focus on parenting skills. One of the workbooks is designed for parents not currently living with their children.

#### Long-Term Tailored Support for the Whole Family

Seven interventions offered longer-term support (at least 1 year long) for families with parental mental illness, and often involved case management and collaboration with other agencies. The *Invisible Children's Project* (20) is mandated as part of a child welfare plan in the U.S. and involves case management for the whole family. *Family Options* (64) is an intervention in the U.S. where Family Coaches are assigned to a family to provide many types of support, including emotional support, advocacy, and goal setting. These Family Coaches can be contacted 24 h a day in the case of an emergency. *Integrated Family Treatment* (72) in the U.S. offers a range of home-based services to families including psychoeducation and signposting to other forms of support. *VIA family* (94) in Denmark assigns families a case manager, and offers a range of supports including psychoeducation, *Triple P* (99), advocacy, social support, and liaison with schools. *Preventative Basic Care Management (PBCM)* (84) in the Netherlands also assigns families a case manager and coordinates the services involved in the families' care. The *BROSH program* (53) lasts 2 years and is a collaboration from child welfare, child mental health and adult mental health services is Israel. It consists of weekly home meetings either with the parent or the whole family where parents learn about child development, mentalizing skills, and can get help with financial issues. The children are also offered individual psychotherapy. The *Godparents programme* (71) takes a different approach, in which lay people are trained to perform the godparent role in Switzerland. They are assigned to a family for at least 3 years and act as another adult figure for the child and social support for the parent.

#### Groups for Parents With Mental Illness

Five interventions were designed as groups for parents with mental illness. *Living with Under Fives* (78) and *FWA Newpin* (70) are both designed for parents with children up to 5 years old and provide a space for the parent and child to play together alongside other families. *Living with Under Fives* also offers components on psychoeducation, parenting skills, budgeting, and links parents with other agencies. Shor et al. (89) describe a long-term therapeutic group for parents where they can raise parenting issues and give each other advice. The primary aim of *Young SMILES* (97) is to improve the quality of life of children affected by parental mental illness by teaching children about mental illness, recognizing stress, and accessing support networks. It includes a parent group that has components on supporting their children and successful family communication. *SEEK* (86) was developed as a compulsory part of treatment for parents with mental illness whose children are currently in inpatient treatment. It involves psychoeducation on mental illness, talking to children about mental illness, and family stress.

#### Family Therapy

Three interventions were focused on providing family therapy. The *Think Family Whole Family Programme* (90) is based on the Meriden Family Programme (102), which is a behavioral family intervention that teaches communication and problem-solving skills. The *Think Family Whole Family Programme* adds further elements about parental mental illness. The *Child Resilience Program* (56) provides family therapy with separate parent and child groups, as well as sessions on psychoeducation, parenting skills, and building resilience. Becker et al. (60) briefly describes a counseling and support service for the whole family.

### Evaluations of Interventions

Twenty-three out of the 38 included studies of interventions had some kind of quantitative evaluation of parent or child outcomes, and 13 studies involved a qualitative evaluation of acceptability from the parents. Eight studies had both a quantitative and qualitative evaluation.

Table 3 lists the demographic details of participants. All interventions had more female participants than male. In all studies apart from Wolfenden (93) and Strand and Meyersson (68), in which every participant had a psychotic diagnosis, the proportion of participants with a psychotic diagnosis ranged between 0 and 42.5%, or was unknown. There were in total at least 53 participants with a psychotic diagnosis in the studies with a quantitative evaluation, and at least 60 in the studies with a qualitative evaluation.

**Table 3:** Participant characteristics in 23 completed evaluations of included interventions.

**Intervention name**	**No. of parent participants**	**Percentage with psychotic diagnosis**	**Age of parents (mean, standard deviation or range)**	**Percentage of mothers**	**Ethnicity of parents**	**Marital or living status of parents**	**Education of parents**	**Employment of parents**	**Age of children (mean, standard deviation or range)**	**Percentage of daughters**	**Number of children in family**	**Percentage of children living with parents**
BROSH program (53)	11	36.4%	Mean = 39.2 Range = 32–57	*Unknown*	*Unknown*	27.3% single 27.3% divorced 45.4% married	*Unknown*	57% unemployed	Range = 2 months−11.5 years	*Unknown*	*Unknown*	*Unknown*
Family options (63)	22	4.6%	Mean = 36 SD = 8.3	100%	77.2% White 9.1% Black 9.1% Hispanic 4.6% Asian	36.4% lived with a significant other	More than 80% completed high school	18% part or full-time employed	*Unknown*	52%	Mean = between 2 and 3 SD = 1.3 Range = 1–5	88.5% of children lived with parents
Family Talk (67)	66	13.6%	*Unknown*	80.3%	*Unknown*	32% single	*Unknown*	*Unknown*	Median = 12	*Unknown*	*Unknown*	*Unknown*
Family Talk (68)	8	100%	*Unknown*	75%	*Unknown*	*Unknown*	*Unknown*	100% unemployed and unable to work	Range = 8–15	57.1%	*Unknown*	86% lived with at least one parent 14% placed in foster care
Family Talk (65)	37	0%	*Unknown*	*Unknown*	*Unknown*	*Unknown*	*Unknown*	*Unknown*	Mean = 10.41 SD = 2.66	*Unknown*	*Unknown*	*Unknown*
Integrated family treatment (72)	8	*Unknown*	Range = 20–41	100%	100% Caucasian	37.5% not living with partner 62.5% married or living with partner	62.5% at least high school education	*Unknown*	*Unknown*	*Unknown*	Range = 1–4	*Unknown*
KidsTime (74)	5	*Unknown*	*Unknown*	100%	*Unknown*	*Unknown*	*Unknown*	*Unknown*	*Unknown*	*Unknown*	*Unknown*	*Unknown*
KopOpOuders (22)	48	6.3%	Mean = 37 SD = 6.8	85.4%	90% Dutch 10% Belgian, Turkish or Danish	58% dual parent families 56% married	42% intermediate education 27% higher education	52% employed	Mean = 6.7 SD = 5.3	*Unknown*	83% of parents had 1 or 2 children	*Unknown*
Let's talk about children (75)	39	42.5%	Mean = 39.9 Range = 26–62	94.9%	*Unknown*	51.2% single parent household	*Unknown*	*Unknown*	Mean = 9.5 Range = 6 months−18 years	*Unknown*	Mean = 1.8 Range = 1–5	*Unknown*
Let's talk about Children booklet (77)	19	0%	Mean = 42.9 Range = 34–60	89.5%	94.7% born in Australia 5.3% born overseas	26.3% single 57.9% married or living together 15.8% separated or divorced	5.3% primary education 42% intermediate education 52.7% higher education	*Unknown*	*Unknown*	*Unknown*	Mean = 1.8	84.2% lived full time with children 10.6% lived with children more than half the time 5.2% lived with children less than half the time
Parenting internet intervention (80)	60	13.3%	Mean = 37 SD = 7	100%	*Unknown*	*Unknown*	*Unknown*	*Unknown*	*Unknown*	*Unknown*	*Unknown*	*Unknown*
Parenting with success and satisfaction workbooks (82)	26	7.7%	Range = 21–52	76.9%	*Unknown*	42% unmarried 19% married 39% divorced/widowed	54% primary education 42% intermediate education 4% higher education	42% employed	*Unknown*	*Unknown*	35% had 1 child 65% had 2–4 children	69% were legally responsible for their child 12% were legally responsible with a foster poster 19% were not legally responsible for their child
Preventive basic care management (PBCM) (85)	99	*Unknown*	*Unknown*	87.9%	33% Dutch 19% Moroccan 15% Turkish 14% Surinamese 7% Netherland Antilles 12% other	46% single parent family	*Unknown*	*Unknown*	Mean = 6.08	45%	Mean = 2.13	*Unknown*
SEEK (86)*	26	*Unknown*	Mean = 37.1	92.3%	*Unknown*	34.6% single 53.8% married 11.6% divorced/separated 65.4% living with a partner	3.4% primary education 65.3% intermediate education 30.7% higher education	*Unknown*	Mean = 5.92	46.2%	*Unknown*	*Unknown*
Strengths based parenting programme (unnamed) (87)	4	25%	Mean = 36.75 Range = 23–48	75%	100% Anglo-Australian	*Unknown*	*Unknown*	*Unknown*	Mean = 9.6 Range = 2–21	*Unknown*	50% had 1 child 25% had 2 children 25% had 8 children	*Unknown*
The lighthouse (leuchtturm) parenting programme (88)*	5	0%	*Unknown*	100%	*Unknown*	*Unknown*	*Unknown*	*Unknown*	*Unknown*	*Unknown*	*Unknown*	*Unknown*
Therapeutic group (unnamed) (89)	35	14.3%	Mean = 43	45.7%	*Unknown*	50% divorced or separated	*Unknown*	*Unknown*	Mean = 2.7 Range = 1–9	*Unknown*	*Unknown*	*Unknown*
Triple P (91)*	42	0%	Mean = 37 SD = 5.1	83.3%	*Unknown*	70% married or living with partner 17% single/separated/ divorced 13% Unknown	*Unknown*	*Unknown*	Mean = 6 SD = 2.7	43%	61.5% had one child 27% had two children 11.5% had three children	*Unknown*
Triple P + mental health components (36, 103)	86	4.7%	Mean = 32.6 SD = 6.4	90.7%	93% Not aboriginal or Torres Strait 7% Aboriginal or Torres Strait	38% single 62% married or living with partner	*Unknown*	*Unknown*	Mean = 4.9	38%	*Unknown*	*Unknown*
Triple P self-help workbook (93)	10	100%	Mean = 33 Range = 26–48	100%	80% White British 10% Black other 10% Chinese	90% sole parent household 10% cohabiting	30% primary education 10% intermediate education 60% higher education	10% employed part-time 90% unemployed and not able to work	Mean = 8 Range = 4–10	40%	Mean = 2 Range = 1–5	*Unknown*
Tuning into kids (38)	8	12.5%	*Unknown*	87.5	*Unknown*	*Unknown*	*Unknown*	*Unknown*	*Unknown*	*Unknown*	*Unknown*	*Unknown*
You are okay (95)	41	*Unknown*	Mean = 43.9	85.4%	87.8% born in the Netherlands	51.2% single parent family	26.8% primary education 63.4% intermediate education 9.8% higher education	53.7% unemployed	Mean = 14.1	38.2%	*Unknown*	*Unknown*
Young SMILES (97)	33	9.1%	*Unknown*	90.9%	91% White British 6% Asian 3% *Unknown*	81.8% unmarried	81.8% intermediate education 12.2% higher education 3% *Unknown*	96.9% unemployed	Mean = 10.6	60%	*Unknown*	100% of children lived with parents

Table 4 lists the studies that contained completed evaluations or protocols for evaluations, and reports their design, outcome measures used, and qualitative results.

**Table 4:** Design and results of 28 completed evaluations or protocols for evaluations of included interventions.

**Intervention name**	**QUANTITATIVE EVALUATION**		**QUALITATIVE EVALUATION**	
	**Design**	**Quantitative results**	**Data collection and analysis**	**Qualitative results**
***Studies with both quantitative and qualitative evaluations***				
Let's talk about children (75)	Quasi-experimental	– Parenting ◦ Parenting stress scale – Parent psychosocial ◦ General functioning index of MFAD	Semi-structured interviews Interpretative phenomenological analysis and thematic analysis	• Insight ◦ Parents commented they focused on their child more after LT ◦ Parents felt they family was more connected after LT • Normalizing ◦ LT gave parents more confidence in their own parenting • Family communication ◦ Families talked about PMI more after LT • Clinician support for the parenting role ◦ One parent said her case manager now better sees her in the context of her family • Additional support required • Parents saw LT as the start of a conversation and identified the next stages including helping their children to regulate their emotions
Let's talk about children booklet (77)	Within group pre-post analysis	– Parenting ◦ Parenting self-agency measures, Parenting and mental illness scale *No significance testing*	Semi-structured interviews Thematic analysis	• General feedback regarding the resource ◦ Parents felt they could relate to the resource ◦ Some parents felt the resource could be upsetting ◦ The booklet helped with asking for support • How the parents used the resource ◦ The resource helped parents feel they could start a conversation with their child about PMI ◦ One parent questioned whether it was important to have conversations about PMI • Recommendations for dissemination ◦ The resource is useful for parents at all stages of their illness • One parent suggested that it would only work for those who had accepted their diagnosis
The lighthouse (leuchtturm) parenting programme (88)	Within group pre-post analysis	– Parenting ◦ EBI *No significance testing*	*Unknown*	• Parents enjoyed the mentalization metaphors • Parents enjoyed the group format and speaking to other parents with mental illness • Some parents asked for longer and more sessions • Parents reported their stress levels decreasing • Parents reported their parenting self-efficacy increasing
Parenting with success and satisfaction workbooks (83)	Non-randomized controlled trial	– Parenting ◦ TOPSE – Parent psychosocial ◦ PES – Parent quality of life ◦ WHOQOL-BREF, EUROQOL-VAS^*^	Semi-structured interviews *Unknown analysis*	• Parents could identify relevant support systems following intervention • One parent said she felt she had made progress in her role as a mother
Triple P + mental health components (36, 103)	Within group pre-post analysis	– Child behavior ◦ ECBI^*^ – Parenting ◦ Parenting scale^*^	Semi-structured interviews Thematic analysis	• Being in a group with others with mental illness ◦ Knowing others also had a mental illness reduced anxiety ◦ Parents felt they had similar experiences to others in the group and felt understood • Focus on child development and parenting with a mental illness ◦ Parents felt they learnt techniques on how to handle their child's behaviors ◦ Parents could identify their own triggers so felt more in control ◦ Parents felt they understood their children more after Triple P • The home visits • Parents felt the home visits at the end of the intervention helped embed the learning from Triple P
Triple P self-help workbook (93)	Within group pre-post analysis	– Parenting ◦ Parenting tasks checklist, Parenting scale^*^, Parenting and family adjustment scales^*^ – Parent psychosocial ◦ Psyrats^*^, DASS-21, PANSS, Calgary Depression Scale, PSP^*^, WEMBWBS^*^ – Child behavior ◦ ECBI^*^, SDQ^*^	Semi-structured interviews Interpretative phenomenological analysis	• The discovery of self and lost possibilities ◦ Parents felt positive about taking part in Triple P ◦ Parents spoke about the relationship between mental health and parenting ◦ Parents felt they were more in control after Triple P • The transition to appropriate parenting ◦ Parents felt their parenting had improvement after Triple P e.g., less screaming and more open communication with their child ◦ Parents thought their children were happier after Triple P and that family life was better • Parents took more pride from their role as a parent after Triple P
Tuning into kids (38)	Within group pre-post analysis	– Parenting ◦ Parents concerns questionnaire^*^ – Parent psychosocial ◦ K10, DERS, PESQ	Open-ended questionnaire Conventional content analysis	• Parents felt comfortable in the group format • Some parents felt they were more skilled in their parenting at Tuning into Kids • Some parents identified communication with their child was better • One parent said she felt she could help her daughter with her anxiety more
Young SMILES (97)	Feasibility RCT	– Child quality of life ◦ PedsQL, KIDSCREEN, CHU9D – Child psychosocial ◦ RCADS – Child behavior ◦ SDQ – Parenting ◦ Mental health literacy questionnaire, Parenting Scale, PSI *No significance testing*	Semi-structured interviews Thematic analysis	• Intervention coherence ◦ Some parents felt there was not enough focus on them as a parent • Affective attitude ◦ Parents were keen for their child to understand PMI ◦ Parents felt hopeful for the future after attending Young SMILES ◦ Some parents felt comfortable with the group approach and some didn't like it • Burden ◦ Parents felt anxious about going to the group ◦ Some parents felt pressured to attend the group • Ethnicity ◦ Some parents valued separate parent and child groups and some wished they had been with their children ◦ Parents enjoyed the setting of the Young SMILES intervention • Opportunity costs ◦ One parent interpreted Young SMILES as claiming her mental illness was damaging her child ◦ One parent said the assessment was too invasive and her mental health declined as a result • Perceived effectiveness ◦ Parents felt their children were coping better after Young SMILES and that the family environment was more relaxed ◦ Parents enjoyed being in a group with others who had similar experiences • Self-efficacy ◦ Parents spoke highly of the facilitator and the non-judgmental nature of the group • Parents felt respected in the group
***Studies with only a quantitative evaluation***				
BROSH Program (53)	Within group pre-post analysis	– Parent psychosocial ◦ CANS subscale—impact on caregiver – Child psychosocial ◦ CANS subscale—affect regulation *No significance testing*		
Child talks+ (57)	Protocol Full RCT	– Child quality of life ◦ KIDSCREEN-27, PEDS – Child psychosocial ◦ READ, GSQ-APMI, Children's mental health literacy scale – Child behavior ◦ SDQ – Parenting ◦ Parent-child communication scale, PSCS		
CHIMPS intervention (59)	Protocol Full RCT	– Child psychosocial ◦ Schedule for affective disorders and schizophrenia for school aged children, Youth self-report, Children global assessment scale – Child behavior ◦ CBCL – Child quality of life ◦ KIDSCREEN – Parent psychosocial ◦ BSI, Health questionnaire, Global assessment of relative functioning, Oslo social support questionnaire – Parent quality of life ◦ EQ-5D – Parenting ◦ FB-A		
Family options (63)	Within group pre-post analysis	– Parent psychosocial ◦ Global Severity Index of BSI^*^, Posttraumatic Stress Symptom Scale, SF-8, MOS-SSS		
Family Talk (65)	Non-randomized controlled trial with healthy control group	– Child behavior ◦ CBCL^**^, SDQ^**^ – Parenting ◦ Knowledge about mental illness questionnaire^**^		
Family Talk (66)	Protocol Full RCT	– Child behavior ◦ SDQ – Child psychosocial ◦ RCADS, SCARED-5, CYRM-12 – Parent psychosocial ◦ BASIS-24, CSE		
Integrated family treatment (72)	Within group pre-post analysis	– Parenting ◦ HOME, Parent Stress Inventory – Parent psychosocial ◦ BSI – Child quality of life ◦ Lehman Quality of Life interview *No significance testing*		
KopOpOuders (22)	Within group pre-post analysis	– Parenting ◦ Parenting Scale^*^, OOO^*^ – Child behavior ◦ SDQ		
Parenting internet intervention (80)	Full RCT	– Parenting ◦ PSCS^**^, HFPI^**^, MOS-SSS, Family Coping Inventory		
Preventive basic care management (PBCM) (85)	Full RCT	– Parenting ◦ HOME, Parenting skill subscale of FFQ^**^, Parenting Daily Hassles – Child behavior ◦ SDQ		
SEEK (86)^*^	Non-randomized controlled trial	– Parenting ◦ EBI^*^ – Parent psychosocial ◦ HSCL-25 – Child behavior ◦ CBCL		
Triple P (91)^*^	Non-randomized controlled trial with healthy control group	– Parent psychosocial ◦ DASS-21^**^ – Parenting ◦ EFB-K ◦ PEV – Child behavior ◦ SDQ^**^		
Triple P + CBT (37)	Protocol Full RCT	– Child behavior ◦ CBCL – Child psychosocial ◦ Kinder-DIPS – Parent psychosocial ◦ DIPS, BSI, PID-5-BF – Parenting ◦ EFB, ESF, Child knowledge about mental disorders – Child quality of life ◦ KIDSCREEN-10 – Parent quality of life ◦ EUROQOL, AQoL-8D		
VIA family (94)	Protocol Full RCT	– Child behavior ◦ CBCL - Child psychosocial ◦ CGAS, Days absent from school – Parenting ◦ FAD, HOME		
You are okay (95)	Quasi-experimental	– Child behavior ◦ SDQ^*^ – Child psychosocial ◦ Self-perception profile for adolescents, COMPI specific cognitions, NRI-BSV – Parent psychosocial ◦ SSL-12-I – Parenting ◦ Perceived parental competence, Parental involvement with child's treatment, Parenting Scale		
***Studies with only a qualitative evaluation***				
Family Talk (67)			Open-ended questionnaire *Unknown analysis*	• Important for parent's recovery that the children understood how they had experienced their illness • Relationship with partner strengthened post Family Talk • Communication was easier post Family Talk • Parents felt they learned to focus on children more
Family Talk (68)			Semi-structured interviews Qualitative content analysis	• Information ◦ Family Talk improved family members' knowledge about PMI ◦ FT meant the child knew who to turn to if their parent became ill • General parenting and child support ◦ Some parents felt they had received good advice on parenting ◦ Some parents felt that FT had not given them any specific support or made any concrete changes • Communication ◦ Before FT, parents hesitated to talk about PMI ◦ Some parents felt FT allowed them to communicate with their child about PMI, and others still found it too difficult to talk about • Understanding ◦ Family members felt their understood each other's experiences better after FT ◦ Parents who did not have custody of their children felt FT gave them an insight in their children's daily lives • Structure ◦ Parents appreciated that their child was able to talk to the professional delivering the intervention ◦ Parents appreciated the structure of the intervention and that the professional followed a manual ◦ Some parents asked for a more holistic structure, where their illness wasn't the focus, and other family problems could be discussed
KidsTime (74)			Semi-structured interviews Thematic analysis	• Aims and impact ◦ Parents felt they could communicate about PMI to their child ◦ Parents gained more awareness about how PMI affected their child ◦ Parents enjoyed being in a group of others with similar experiences ◦ Parents felt their relationship with their child has improved, and that they feel more confident in their parenting role • Nature of referral process ◦ Parents appreciated that they were referred by the school in contrast to being referred by a health or social care system • Need for extended support ◦ Parents wanted more support for their children in schools
Strengths based parenting programme (unnamed) (87)			Written reflections and semi-structured interviews Thematic analysis	• Parents felt the programme helped them communicate effectively with their child • Parents felt they could relax a bit more during difficult parenting moments • Parents felt their understood their emotions better and could help their children to do so too
Therapeutic group (unnamed) (89)			Open-ended questionnaire Grounded theory	• Overcoming difficulties to connect to the children and maintain relationships with them ◦ Parents provided suggestions to each other on how to maintain contact with their child ◦ Parents felt comfortable in the group to share these difficulties
				• Speaking with the child about the mental illness ◦ Group members discussed whether or not to tell their child about their mental illness and how to do this in an age appropriate way • Improving parenting skills and developing the role of a parent ◦ Parents expressed insecurities in their own parenting ◦ Group members gave each other advice on setting boundaries and discipline • Hopes and fears regarding parenting ◦ Parents spoke about their goals which including meeting child more often, developing a good relationship with their child, and taking more responsibility for their child

*AQoL-8D, assessment of quality of life; BASIS-24, behavior and symptom identification scale 24; BSI, brief symptom inventory; CANS, child and adolescent needs and strengths; CBCL, child behavior checklist; CGAS, children's global assessment scale; CHU9D, child healthy utility 9D; CSE, coping self-efficacy questionnaire; CYRM-12, child and youth resilience measure 12; DASS-21, depression anxiety and stress scales short form; DERS, difficulties in emotional regulation scale; DIPS, diagnostic interview of mental disorders for parents and children; EBI, Eltern-Belastungs-Inventar; ECBI, Eyberg child behavior inventory; EFB, erziehungsfragebogen; ESF, elternstressfragebogen; FAD, family assessment device; FB-A, allgemeiner familienfragebogen; FFQ, family functioning questionnaire; GSQ-APMI, guilt and shame questionnaire for adolescents of parents with mental illness; HFPI, healthy families parenting inventory; HOME, home observation for measurement of the environment; HSCL-25, Hopkins symptom checklist-25; K10, Kessler psychological distress scale; MOS-SSS, medical outcomes study, social support survey; NRI-BSV, network of relationships inventory-behavioral systems version; OOO, Ouderlijke Opvattingen over Opvoeding; PANSS, positive and negative syndrome scale; PEDS, parents' evaluations of developmental status; PES, psychological empowerment scale; PESQ, parents emotional style questionnaire; PEV, positives elternverhalten; PID-5-BF, personality inventory for DSM-5-brief form; PSI, parent stress index; PSCS, parenting sense of competence scale; PSOC, parenting sense of competence; PSP, personal and social performance scale; PSYRATS, psychotic symptom rating scales; RCADS, revised child anxiety and depression scale; READ, resilience scale for adolescent; SCARED-5, screen for child anxiety related disorders; SCORE-15, systematic clinical outcome and routine evaluation; SDQ, strengths and difficulties questionnaire; SF-8, short form-8; SSL-12-I, Dutch social support list-interactions; TOPSE, tool to measure parenting self-efficacy; WEMBWBS, Warwick Edinburgh mental well-being*.

* *For sig. improvement with intervention group pre vs. post*.

** *For sig. improvement between intervention and control group post intervention*.

#### Quantitative Evaluations

Out of the 23 quantitative evaluations, 11 had a control group and only eight randomly assigned the participants to the control or intervention group. Out of these eight randomized control trials (RCTs), five were protocols. The three completed RCTs evaluated *PBCM*, (85), the *Parenting Internet Intervention* (80), and *Young SMILES* (97). The number of participants in completed studies ranged from eight to 99.

Most interventions had an outcome measure for both the parent and the child. The interventions that only involved the use of a measure for the parent included *Family Options* (63), *Let's Talk about Children* in both the face-to-face and booklet format (75, 77), *Parenting with Success and Satisfaction* (82), *Tuning into Kids* (38), The *Lighthouse (Leuchtturm) Parenting Programme* (88), and the *Parenting Internet Intervention* (80). There was very little consistency in terms of which outcome measures were used. For example, while both *Child Talks*+ and *Let's Talk about Children* aimed to enable the parent to explain their mental illness to their child, *Child Talks*+ included six child outcome measures and two parent measures on communication and self-efficacy (57) while *Let's Talk about Children* only used measures on parenting stress and family functioning (75). There was also variation in which measure each study had seen an improvement. For example, *You are Okay* (95) and *Family Talk* (65) appeared to have an impact on child behavior, whilst *KopOpOuders* and *Mental Health Triple P* appeared to have improved parenting skills.

Randomized controlled trials are the gold standard for the assessment of effectiveness, with non-randomized trials or trials without a control group being susceptible to a range of sources of bias (104). Three RCTs were included in this review. *Young SMILES* did not conduct significance testing or report effect sizes as it was a feasibility trial. The other two RCTs, *Preventative Basic Care Management* and the *Parenting Internet Intervention* both showed improvement on parenting measures of skills and self-efficacy (80, 85). *Preventative Basic Care Management* reported improvement on the parenting subscale of the Family Functioning Questionnaire (85). The *Parenting Internet Intervention* showed improvement on two measures of parenting: Healthy Families Parenting Inventory and Parenting Sense of Competence Scale, but not on the Medical Outcomes Study—Social Support Survey (80). The *Parenting Internet Intervention* did not include any child outcome measures (80). *Preventative Basic Care Management* measured child behavior using the Strengths and Difficulties Questionnaire, but did not find any significant differences between the intervention and control group following the intervention (85).

#### Qualitative Evaluations of Acceptability

Table 4 provides a narrative summary of the qualitative results of the included reports. Thirteen studies involved a qualitative evaluation with eight reporting themes. Parents reported in eight out of 13 studies that they felt they could communicate more easily with their children about parental mental illness after receiving the intervention. This included two studies reporting on the *Family Talk* intervention (67, 68), both studies on *Let's Talk About Children* (75, 77) and *KidsTime* (74), in which the aim of the intervention is to enhance communication. Parents in five out of 13 studies felt their parenting had improved following the intervention, which includes four studies in which the aim was to enhance parenting skills, two *Triple P* studies (93, 103), *Tuning into Kids* (38), the *Lighthouse (Leuchtturm) Parenting Programme* (88), as well as Shor et al.'s (89) therapeutic group. Parents in seven out of 13 studies reported that they understood, and could focus, on their children's needs more. Parents in one evaluation of *Family Talk* said that the intervention played an important part in their recovery (67).

For the six interventions that were held in a group format, parents all commented on how they enjoyed being in a group with other parents who have experienced similar difficulties, although some of the parents who took part in *Young SMILES* reported they felt anxious and pressured about attending. The parents in *Mental Health Triple P* also commented that they enjoyed the home visits (103).

These results suggest that most interventions have a good level of acceptability to parents, and there was also appreciation for different intervention formats including groups and home visiting.

Parents in four studies highlighted potential improvements on structure of the intervention. In the *Family Talk* intervention for parents with psychosis, parents said they would have preferred an intervention where their illness was not the focus (68). Some parents who received the *Let's Talk about Children* booklet found it upsetting (77). In *Young SMILES*, parents felt there was too much emphasis on their child and not enough on them as a parent, and one parent reported that the focus on her mental illness felt damaging (97). Parents in the *Lighthouse (Leuchtturm) Parenting Programme* stated they wanted a higher number of sessions which were longer in duration (88). In two out of 13 studies, parents spoke about the next stages, which included wanting more support for their children in schools (74) and wanting to help their child regulate emotions better (75).

## Discussion

### Summary of Evidence

This scoping review involved a systematic search of relevant databases and other sources to establish what a parenting intervention for parents with psychosis might look like. The three aims of this review were to determine (1) what parenting interventions were available for parents with psychosis, (2) what components these interventions provided, and (3) what kinds of evaluations had been undertaken, and what they showed in terms of outcomes. Thirty-eight studies were included which described 34 interventions.

#### What Parenting Interventions Are Available for Parents With Psychosis?

Thirty-four interventions were described, of which most were designed for either parents with mental illness or parents with severe mental illness. When parents with psychotic diagnoses were included in these interventions, there were often in the minority compared to parents with other diagnoses. Both researchers (105) and parents diagnosed with mental illness (106) have recommended the use of diagnostic-specific groups, and recently, RCTs of parenting interventions for parents with anxiety (42) and with borderline personality disorder (43) have been conducted, and report promising results. In this review, only two interventions focused solely on parents with a psychotic diagnosis, and both had a sample size of 10 participants or fewer. These were *Family Talk* (68) and *Triple P* (93), both of which were unchanged from their usual delivery format. It may be the case that parents with psychosis would benefit from specific additions to parenting interventions, like safety planning for acute episodes (107), or a focus on regaining self-confidence during periods of stabilization (108).

Parents with mental illness often want their family to be involved in their treatment (21), and parenting can be a valued part of one's personal recovery (27). Reflecting this desire, most interventions in this review were designed either for the parent with a mental illness and their child, or for the whole family, which typically included the parent with a mental illness, their children, their partner, and sometimes additional family members. When interventions were designed solely for the parent, they were often delivered in a group format. Parents with mental illness can often face social isolation (14), and an intervention in a group setting could be one way of alleviating this. Parents with psychosis, specifically asked for a group intervention in order to be able to meet others in a similar situation, share parenting tips, and find social support (109). However, parents in the *Young SMILES* intervention found that attending a group can also be anxiety provoking (97).

Despite the fact that these parents can face poor social and emotional support, only a few interventions incorporated peer support, where someone who has also experienced poor mental health is involved in delivering the intervention (110). Having parent peers involved in delivering parenting interventions may help alleviate the lack of social support, and could also help to reduce the stigma felt by parents (111).

When considering the availability of interventions, it is important to note that geography is one of the biggest limiting factors in terms of which interventions parents can access. The 38 studies included in this review came from 14 countries, the majority of which were from Australia, who have also been a leader in policy advancement for parents with mental illness and their children for the last 20 years (112). As well as integrating interventions in mental health and social care services, the parenthood status of patients must be identified. This has been done well in Norway where, alongside the *Child Talks*+ intervention, an assessment form has also been implemented to improve recording and identification of patients' dependants (113). It is not enough for these interventions to be developed and tested, they need to be recommended in policy and made available to the parents who would benefit from them.

#### What Are the Components of These Interventions?

The interventions identified in this review were grouped into five categories, depending on the cluster of their components. It is important to consider which of these five categories of interventions best address the needs of parents with psychosis.

The largest group, which consisted of 11 interventions, had a focus on improving parenting skills, and the one RCT, Kaplan et al.'s (80) *Parenting Internet Intervention*, demonstrated improvement on measures of parenting satisfaction and coping skills. Parents with psychosis have demonstrated difficulties in reflective functioning and parental sensitivity (6, 14, 114), and this is particularly true for individuals with a higher severity of illness (12, 115). However, parents with psychosis and their families may need more support that goes beyond just addressing parenting skills.

The children affected by parental mental illness have expressed a desire for their parent's symptoms to be explained to them (19, 21), and the second largest group of interventions was developed in response to this need. Eight interventions had a focus on explaining mental illness to the children. Often, they also included psychoeducation about the effects of parental mental illness on the child. Additionally, these interventions provided an opportunity for the children and, sometimes, the parent's partner, to talk about their experiences of parental mental illness. However, psychoeducation about parental mental illness alone may not be sufficient to bring about positive change for the parent or for their child (116). Parents with psychosis who participated in *Family Talk* stated that they wanted less focus on the effects of their illness (68), and parents who had participated in *Young SMILES* stated they wanted more parenting components, and not solely a focus on their children (97).

The third largest group consisted of seven long-term whole family interventions, which typically lasted longer than the other interventions, and were more holistic. These often involve case management, whereby the family receives continuous care from one individual, interagency collaboration and links with other supportive agencies. Often crisis planning for potential relapses is also incorporated, as well as help with other difficulties that affect these families, such as financial issues. An example of one of these interventions is *VIA Family*, which had multiple stages. First the family is introduced to the intervention, then a life history is taken, and the family received psychoeducation. Then *Triple P* is offered and, finally parent and children groups are provided. Throughout the intervention, there are many optional extras, such as psychological treatment for the child's mental health difficulties, advice on finances, and social support for the parent (94).

The needs of parents with psychosis are often complex and diverse. Parents with severe mental illnesses have reported difficulties with practical issues such as finances and household tasks as well as fears about custody loss (25). Parents with psychosis and their families additionally struggle with parenting skills (6, 14, 117), self-confidence (109), and relapse of symptoms and subsequent hospitalization (117). Furthermore, these needs may be different during acute episodes of psychosis and periods of stability (108, 117). Therefore, interventions that solely address parenting skills or aim to explain mental illness to the children of these parents are likely to be insufficient, and more holistic long-term interventions may be the most suitable to address the needs of this group of parents. However, a more complex intervention will come with higher costs. Only *Preventative Basic Care Management* has been subjected to a cost-effectiveness evaluation (118). The authors stated that the intervention was more costly than care as usual, but could not conclude whether it was cost-effective or not (118). Identification of the essential components needed to enhance the well-being of these parents and their families is needed to enable us to implement effective interventions both in terms of psychosocial and economic outcomes.

It is also necessary to note that inpatient facilities in Germany often provide many components described in this study, such as selfcare, peer support, and signposting, as part of routine inpatient treatment (119) and that those receiving the *SEEK* intervention (86) and the *Lighthouse Parenting Programme* (88) will have also benefitted from these elements.

#### What Kinds of Evaluations Have Been Conducted to Determine the Acceptability and Effectiveness of Interventions for Parents With Psychosis and What Do They Show?

Parenting interventions for parents with mental illness are relatively new, and as such have an emerging evidence base. Around two-thirds of the interventions described in this review had been evaluated in some way, and only eight of these evaluations were RCTs, with only three having results available. One of these RCTs, *Young SMILES* (97), did not conduct significance testing since it was a feasibility trial. The other two, *Preventative Basic Care Management* (85) and Kaplan et al.'s (80) *Parenting Internet Intervention*, demonstrated significant differences between the parents in the intervention and control groups on measures of parenting. Therefore, it seems there is initial evidence that parenting interventions for parents with mental illness can improve aspects of parenting, such as skills and self-efficacy.

Children of parents with any kind of mental health diagnosis are more likely than children without parental mental illness to exhibit internalizing and externalizing problems (16) and are at risk of developing a mental health problem (120, 121). While, in theory, enhancing parenting skills should improve the child's quality of life and later psychosocial health (122), it is nevertheless still important to assess changes in children's functioning following such intervention. The RCT with the longest follow-up in this review was *Preventative Basic Care Management* (85), and did not report any difference in child behavior between the intervention and control group after 18 months of intervention. There is therefore, currently a lack of evidence demonstrating the effectiveness of parenting interventions in producing positive outcomes for the children of parents with mental illness. The longest two RCTs that are currently taking place are *VIA Family* (94) and *Triple P* combined with CBT (37), and it will be noteworthy to see if these interventions have any impact on children's functioning at follow-up.

Thirteen studies involved a qualitative evaluation of a parenting intervention. Most studies reported positive comments made by parents on intervention content and format, indicating that most interventions have a good level of acceptability. However, some parents who received the *Let's Talk about Children booklet* found it upsetting (77), which highlights the importance of parents with mental illness being supported by a professional during the delivery of parenting interventions. Parents in the *Family Talk* intervention and *Young SMILES* wanted less focus on their mental health (68, 97), and parents in *Young SMILES* also wanted more focus on them as a parent rather than solely on their child (97). These results suggest that interventions should be careful not to stigmatize or blame parents, and should recognize the centrality of their identity as a parent (27).

## Strengths and Limitations

This review has updated the results from the reviews conducted by Schrank et al. (47) and Suarez et al. (48), which identified fifteen and nine interventions, respectively. In contrast to Schrank et al. (47) and Suarez et al. (48), this review did not set a limit for what proportion of the study sample needed a psychotic diagnosis, and included interventions that had not yet been evaluated. Additionally, many of the interventions included in this review have been published in the 5 years since Schrank et al. (47) and Suarez et al. (48) conducted their reviews. Since this review did not solely include interventions which had been tested with a certain proportion of parents with a psychotic disorder, it identified many interventions that could be helpful for parents with psychosis and their families.

Scoping reviews do not necessarily need a quality assessment (50). However, one limitation of this review is that the lack of quality assessment means the results of the studies included in this review are not contextualized alongside an assessment of their risk of bias. The main limitation of this review is that it only included papers that are published in English or German. Fifty reports were rejected at full-text review due to being written in another language, and it is likely that some would have been eligible for inclusion in this review. Another limitation relates to how we identified the components of each intervention, in which we only extracted the components that had been described in the report of each study, some of which did not always contain much detail. It may well be the case, therefore, that some interventions included more components than indicated in Table 2.

## Future Directions

Future research needs to investigate which components are the most effective in improving outcomes for both the parent and the child. The needs of parents with psychosis and their families are complex, and it is not sufficient for interventions to aim solely to enhance parenting skills or explain mental illness to their children. Only two interventions in this review were conducted exclusively with parent participants with a diagnosis of psychosis (68, 93), and yet they had been unchanged from their usual delivery format and therefore not tailored toward the needs of parents with psychosis. Interventions must attempt to address practical issues, periods of unplanned hospitalization, and parents' own self-confidence and self-efficacy.

When addressing parenting skills, a psychotic diagnosis does predict deficits in social cognitive abilities (45), which affects parents' ability to understand their child's mental states (114). Therefore, parents with psychosis would likely benefit from interventions with a mentalizing component, which was the case in four interventions included in this review (38, 53, 70, 88).

When interventions did include parents with a psychotic diagnosis in their evaluation, they were often in the minority compared to parents with other mental health conditions. Interventions which are designed for parents with any kind of mental illness should endeavor to include more parents with a psychotic diagnosis when evaluating the intervention in order to determine whether these interventions are indeed effective for those with more severe mental illnesses, like psychosis.

It is promising that some of the interventions in this review are currently being tested in an RCT. As well as testing interventions, we must investigate what types of interventions are most effective, in order to produce evidence-based and cost-effective programs.

## Conclusions

Many parenting interventions exist for parents who have experienced mental illness, from which parents with a diagnosis of psychosis and their families may benefit, however no intervention has been developed and evaluated to specifically support parents with psychosis and their families. Five categories of intervention were identified, reflecting their key components. The two largest categories were “talking about parental mental illness” and “improving parenting skills.” The third category described holistic long-term interventions targeting the whole family, and which often involved the provision of a wide range of components, with implications in terms of cost. Of the 34 studies included in this review, only two RCTs provided evidence for the potential effectiveness of the parenting interventions, thereby highlighting the significant evidence gap. In order to help parents who have experienced psychosis and their families, we need to know which components are effective in improving outcomes for both the parent and their children, and whether any psychosis-specific components would benefit these families.

## Data Availability Statement

The datasets presented in this study can be found in online repositories. The names of the repository/repositories and accession number(s) can be found below: https://osf.io/z4rpn.

## Author Contributions

JR: conceptualization, designing the study, selection of sources of evidence, data extraction, data analysis, and writing the manuscript. NS: selection of sources of evidence and data extraction. BM: selection of sources of evidence, data extraction, reviewing, and editing the manuscript. M-LK and FH: selection of sources of evidence. RD: data extraction. LJ and JB: conceptualization, designing the study, supervision, reviewing, and editing manuscript. All authors contributed to the article and approved the submitted version.

## Funding

JR is a D.Phil. student and is funded by Mental Health Research UK.

## Conflict of Interest

The authors declare that the research was conducted in the absence of any commercial or financial relationships that could be construed as a potential conflict of interest.

## Publisher's Note

All claims expressed in this article are solely those of the authors and do not necessarily represent those of their affiliated organizations, or those of the publisher, the editors and the reviewers. Any product that may be evaluated in this article, or claim that may be made by its manufacturer, is not guaranteed or endorsed by the publisher.

## References

[1] American Psychiatric Association. Diagnostic and Statistical Manual of Mental Disorders (DSM-5). American Psychiatric Pub (2013). 10.1176/appi.books.97808904255968723190

[2] Myin-Germeys I Delespaul PA Van Os J. Behavioral sensitization to daily life stress in psychosis. Psychol Med. (2005) 35:733–41. 10.1017/S0033291704004179 15918350

[3] Lally J MacCabe JH. Antipsychotic medication in schizophrenia: a review. Br Med Bull. (2015) 114:169–79. 10.1093/bmb/ldv017 25957394

[4] Seeman M V. Schizophrenia and motherhood. In: Reupert AE Maybery DM Nicholson J Gopfert M Seeman M V, editors. Parental Psychiatric Disorder Distressed Parents and Their Families. 3rd ed. Cambridge: Cambridge University Press (2015). p. 107–16. 10.1017/CBO9781107707559.012

[5] Campbell LE Poon AWC. Parenting challenges for persons with a serious mental illness. Ment Heal Soc Work. (2020). p. 457–74. 10.1007/978-981-13-6975-9_16

[6] Wan MW Salmon MP Riordan DM Appleby L Webb R Abel KM. What predicts poor mother-infant interaction in schizophrenia? Psychol Med. (2007) 37:537–46. 10.1017/S0033291706009172 17076915

[7] Bebbington P. Childhood sexual abuse and psychosis: aetiology and mechanism. Epidemiol Psichiatr Soc. (2009) 18:284–93. 10.1017/S1121189X00000233 20170041

[8] Varese F Smeets F Drukker M Lieverse R Lataster T Viechtbauer W. Childhood adversities increase the risk of psychosis: a meta-analysis of patient-control, prospective-and cross-sectional cohort studies. Schizophr Bull. (2012) 38:661–71. 10.1093/schbul/sbs050 22461484 PMC3406538

[9] Van Wert M Anreiter I Fallon BA Sokolowski MB. Intergenerational transmission of child abuse and neglect: a transdisciplinary analysis. Gend Genome. (2019) 3:247028971982610. 10.1177/2470289719826101

[10] Jungbauer J Stelling K Kuhn J Lenz A. How do mothers and fathers suffering from schizophrenia experience their parenthood? Results from an in-depth interview study. Psychiatr Prax. (2010) 37:233–9. 10.1055/s-0029-1223535 20597037

[11] Abel KM Hope H Faulds A Pierce M. Promoting resilience in children and adolescents living with parental mental illness (CAPRI): children are key to identifying solutions. Br J Psychiatry. (2019) 215:513–5. 10.1192/bjp.2019.118 31190644

[12] Campbell LE Hanlon MC Galletly CA Harvey C Stain H Cohen M. Severity of illness and adaptive functioning predict quality of care of children among parents with psychosis: a confirmatory factor analysis. Aust N Z J Psychiatry. (2018) 52:435–45. 10.1177/0004867417731526 29103308

[13] Ranning A Laursen TM Thorup AAE Hjorthøj C Nordentoft M. Children of parents with serious mental illness: with whom do they grow up? A prospective, population-based study. J Am Acad Child Adolesc Psychiatry. (2016) 55:953–61. 10.1016/j.jaac.2016.07.776 27806863

[14] Abel KM Webb RT Salmon MP Wan MW Appleby L. Prevalence and predictors of parenting outcomes in a cohort of mothers with schizophrenia admitted for joint mother and baby psychiatric care in England. J Clin Psychiatry. (2005) 66:781–9. 10.4088/JCP.v66n0618 15960575

[15] Topor A Andersson G Denhov A Holmqvist S Mattsson M Stefansson CG. Psychosis and poverty: coping with poverty and severe mental illness in everyday life. Psychosis. (2014) 6:117–27. 10.1080/17522439.2013.790070

[16] Johnson SE Lawrence D Perales F Baxter J Zubrick SR. Prevalence of mental disorders among children and adolescents of parents with self-reported mental health problems. Community Ment Health J. (2018) 54:884–97. 10.1007/s10597-017-0217-5 29289984

[17] Luciano A Nicholson J Meara E. The economic status of parents with serious mental illness in the United States. Psychiatr Rehabil J. (2014) 37:242–50. 10.1037/prj0000087 25000119 PMC4152556

[18] Kahng SK Oyserman D Bybee D Mowbray CT. Mothers with serious mental illness: when symptoms decline does parenting improve? J Fam Psychol. (2008) 22:162–6. 10.1037/0893-3200.22.1.162 18266543

[19] Gladstone BM Boydell KM Seeman MV Mckeever PD. Children's experiences of parental mental illness: a literature review. Early Interv Psychiatry. (2011) 5:271–89. 10.1111/j.1751-7893.2011.00287.x 21883973

[20] Hinden BR Biebel K Nicholson J Mehnert L. The invisible children' s project: key ingredients of an intervention for parents with mental illness. J Behav Health Serv Res. (2005) 32:393–408. 10.1097/00075484-200510000-00006 16215449

[21] Wahl P Bruland D Bauer U Okan O Lenz A. What are the family needs when a parent has mental health problems? Evidence from a systematic literature review. J Child Adolesc Psychiatr Nurs. (2017). 30:54–66. 10.1111/jcap.12171 28513062

[22] Van Der Zanden RAP Speetjens PAM Arntz KSE Onrust SA. Online group course for parents with mental illness: development and pilot study. J Med Internet Res. (2010). 12:e50. 10.2196/jmir.1394 21169178 PMC3057319

[23] Park JM Solomon P Mandell DS. Involvement in the child welfare system among mothers with serious mental illness. Psychiatr Serv. (2006) 57:493–7. 10.1176/ps.2006.57.4.493 16603744

[24] Kaplan K Brusilovskiy E O'Shea AM Salzer MS. Child protective service disparities and serious mental illnesses: results from a national survey. Psychiatr Serv. (2019) 70:202–8. 10.1176/appi.ps.201800277 30821211

[25] Dolman C Jones I Howard LM. Pre-conception to parenting: a systematic review and meta-synthesis of the qualitative literature on motherhood for women with severe mental illness. Arch Womens Ment Health. (2013) 16:173–96. 10.1007/s00737-013-0336-0 23525788

[26] Khalifeh H Murgatroyd C Freeman M Johnson S Killaspy H. Home treatment as an alternative to hospital admission for mothers in a mental health crisis : a qualitative study. Psychiatr Serv. (2009) 60:634–9. 10.1176/ps.2009.60.5.634 19411351

[27] Price-Robertson R Obradovic A Morgan B. Relational recovery: beyond individualism in the recovery approach. Adv Ment Heal. (2017) 15:108–20. 10.1080/18387357.2016.1243014

[28] Wyder M Bland R. The recovery framework as a way of understanding families' responses to mental illness: balancing different needs and recovery journeys. Aust Soc Work. (2014) 67:179–96. 10.1080/0312407X.2013.875580

[29] Krumm S Becker T Wiegand-Grefe S. Mental health services for parents affected by mental illness. Curr Opin Psychiatry. (2013) 26:362–8. 10.1097/YCO.0b013e328361e580 23689546

[30] Reedtz C Lauritzen C Stover Y V Freili JL Rognmo K. Identification of children of parents with mental illness: a necessity to provide relevant support. Front Psychiatry. (2019) 9:728. 10.3389/fpsyt.2018.00728 30670987 PMC6333019

[31] Cooklin A. Promoting children's resilience to parental mental illness: engaging the child's thinking. Adv Psychiatr Treat. (2013) 19:229–40. 10.1192/apt.bp.111.009050

[32] Butler J Gregg L Calam R Wittkowski A. Parents' perceptions and experiences of parenting programmes: a systematic review and metasynthesis of the qualitative literature. Clin Child Fam Psychol Rev. (2020) 23:176–204. 10.1007/s10567-019-00307-y 31820298 PMC7192883

[33] Mytton J Ingram J Manns S Thomas J. Facilitators and barriers to engagement in parenting programs: a qualitative systematic review. Heal Educ Behav. (2014) 41:127–37. 10.1177/1090198113485755 23640123

[34] Barlow J Coren E. The effectiveness of parenting programs: a review of campbell reviews. Res Soc Work Pract. (2018) 28:99–102. 10.1177/1049731517725184

[35] Pidano AE Allen AR. The incredible years series: a review of the independent research base. J Child Fam Stud. (2015) 24:1898–916. 10.1007/s10826-014-9991-7

[36] Phelan R Howe DJ Cashman EL Batchelor SH. Enhancing parenting skills for parents with mental illness: the Mental Health Positive Parenting Program. Med J Aust. (2012) 199:S30–3. 10.5694/mja11.11181 25369846

[37] Stracke M Gilbert K Kieser M Klose C Krisam J Ebert DD. CoMPARE family (Children of mentally ill parents at risk evaluation): a study protocol for a preventive intervention for children of mentally ill parents (Triple P, evidence-based program that enhances parentings skills, in addition to gold-standard CBT CBT with the mentally ill parent) in a multicenter RCT-Part II. Front Psychiatry. (2019). 10:54. 10.3389/fpsyt.2019.00054 30873047 PMC6401604

[38] Isobel S Meehan F Pretty D. An emotional awareness based parenting group for parents with mental illness: a mixed methods feasibility study of community mental health nurse facilitation. Arch Psychiatr Nurs. (2016) 30:35–40. 10.1016/j.apnu.2015.10.007 26804499

[39] Beardslee WR Hoke L Wheelock I Rothberg PC Van de Velde P Sawtling S. Initial findings on preventive intervention for families with parental-affective disorders. Am J Psychiatry. (1992). 149:1335–1340. 10.1176/ajp.149.10.1335 1530069

[40] Bee P Bower P Byford S Churchill R Calam R Stallard P. The clinical effectiveness, cost-effectiveness and acceptability of community-based interventions aimed at improving or maintaining quality of life in children of parents with serious mental illness: a systematic review. Health Technol Assess. (2014). 18:1–250. 10.3310/hta18080 24502767 PMC4780907

[41] Reupert AE Maybery DJ. What do we know about families where parents have a mental illness? A systematic review. Child Youth Serv. (2016) 37:98–111. 10.1080/0145935X.2016.1104037

[42] Cartwright-Hatton S Ewing D Dash S Hughes Z Thompson EJ Hazell CM. Preventing family transmission of anxiety: feasibility RCT of a brief intervention for parents. Br J Clin Psychol. (2018) 57:351–66. 10.1111/bjc.12177 29575043

[43] Day C Briskman J Crawford MJ Foote L Harris L Boadu J. Randomised feasibility trial of the helping families programme-modified: an intensive parenting intervention for parents affected by severe personality difficulties. BMJ Open. (2020) 10:1–12. 10.1136/bmjopen-2019-033637 32034024 PMC7045220

[44] Gearing RE Alonzo D Marinelli C. Maternal schizophrenia: psychosocial treatment for mothers and their children. Clin Schizophr Relat Psychoses. (2012). 6:27–33B. 10.3371/CSRP.6.1.4 22453867

[45] Achim AM Ouellet R Roy MA Jackson PL. Mentalizing in first-episode psychosis. Psychiatry Res. (2012) 196:207–13. 10.1016/j.psychres.2011.10.011 22377576

[46] Radley J Grant C Barlow J Johns L. Parenting interventions for people with schizophrenia or related serious mental illness (Review). Cochrane Database Syst Rev. 2020:CD013536. 10.1002/14651858.CD013536.pub2 34666417 PMC8526162

[47] Schrank B Moran K Borghi C Priebe S. How to support patients with severe mental illness in their parenting role with children aged over 1 year? A systematic review of interventions. Soc Psychiatry Psychiatr Epidemiol. (2015) 50:1765–83. 10.1007/s00127-015-1069-3 26091723

[48] Suarez EB Lafrenière G Harrison J. Scoping review of interventions supporting mothers with mental illness: key outcomes and challenges. Community Ment Health J. (2016) 52:927–36. 10.1007/s10597-016-0037-z 27339325

[49] Tricco AC Lillie E Zarin W O'Brien KK Colquhoun H Levac D. PRISMA extension for scoping reviews (PRISMA-ScR): checklist and explanation. Ann Intern Med. (2018) 169:467–73. 10.7326/M18-0850 30178033

[50] Arksey H O'Malley L. Scoping studies: towards a methodological framework. Int J Soc Res Methodol Theory Pract. (2005) 8:19–32. 10.1080/1364557032000119616

[51] Ouzzani M Hammady H Fedorowicz Z Elmagarmid A. Rayyan-a web and mobile app for systematic reviews. Syst Rev. (2016) 5:1–10. 10.1186/s13643-016-0384-4 27919275 PMC5139140

[52] Cohen J. A coefficient of agreement for nominal scales. Educ Psychol Meas. (1960) 20:37–46. 10.1177/001316446002000104

[53] Oppenheim-Weller S Shtark T Aldor R. Families with parental mental illness: studying a home-based intervention program. Child Fam Soc Work. (2021) 26:617–28. 10.1111/cfs.12843

[54] Cowling V Garrett M. Child and family inclusive practice: a pilot program in a community adult mental health service. Australas Psychiatry. (2009) 17:279–82. 10.1080/10398560902840232 19412879

[55] Cowling V Garrett M. A child-inclusive family intervention in a community adult mental health service. Aust New Zeal J Fam Ther. (2012) 33:101–13. 10.1017/aft.2012.1330886898

[56] Gutjahr A. Child Resilience Program an Intervention for Children of Chronically Mentally Ill Parents. Ph.D. thesis, Spalding University (2007).

[57] Reedtz C van Doesum KT Signorini G Lauritzen C van Amelsvoort T van Santvoort F. Promotion of wellbeing for children of parents with mental illness: a model protocol for research and intervention. Front Psychiatry. (2019). 10:606. 10.3389/fpsyt.2019.00606 31572227 PMC6752481

[58] Van Doesum KT Lauritzen C Reedtz C. Child Talks+ Manual. Regional Centre for Child Youth Mental Health Child Welfare. Tromso: The Arctic University of Norway (2020). Available online at: https://uit.no/Content/713118/cache=20210401114628/UiT_RKBU-Nord_A4_manual_ENG_web.pdf (accesesed April 27, 2021).

[59] Wiegand-Grefe S Filter B Busmann M Kilian R Kronmüller KT Lambert M. Evaluation of a family-based intervention program for children of mentally ill parents: study protocol for a randomized controlled multicenter trial. Front Psychiatry. (2021). 11:561790. 10.3389/fpsyt.2020.561790 33551858 PMC7854699

[60] Becker T Kilian S Killian R Lahmeyer C Krumm S. Family needs, children and parenthood in people with mental illness. Eur Psychiatry. (2009) 24:S48. 10.1016/S0924-9338(09)70281-0

[61] Solantaus T Toikka S. The effective family programme: preventative services for the children of mentally ill parents in Finland. Int J Ment Health Promot. (2006) 8:37–44. 10.1080/14623730.2006.9721744

[62] Solantaus T Reupert AE Maybery DJ. Working with parents who have a psychiatric disorder. In: Reupert A Maybery D Nicholson J Gopfert M Seeman M V, editors. Parental Psychiatric Disorder Distressed Parents and Their Families. 3rd ed. Cambridge: Cambridge University Press (2015). p. 238–47. 10.1017/CBO9781107707559.023

[63] Nicholson J Albert K Gershenson B Williams V Biebel K. Developing family options: outcomes for mothers with severe mental illness at twelve months of participation. Am J Psychiatr Rehabil. (2016) 19:353–69. 10.1080/15487768.2016.1231639

[64] Nicholson J Albert K Gershenson B Williams V Biebel K. Family options for parents with mental illnesses: a developmental, mixed methods pilot study. Psychiatr Rehabil J. (2009) 33:106–14. 10.2975/33.2.2009.106.114 19808206

[65] Christiansen H Anding J Schrott B Röhrle B. Children of mentally ill parents - a pilot study of a group intervention program. Front Psychol. (2015). 6:1494. 10.3389/fpsyg.2015.01494 26539129 PMC4611090

[66] Furlong M McGilloway S Mulligan C McGuinness C Whelan N. Family Talk versus usual services in improving child and family psychosocial functioning in families with parental mental illness (PRIMERA—Promoting Research and Innovation in Mental hEalth seRvices for fAmilies and children): study protocol for a randomi. Trials. (2021) 22:1–18. 10.1186/s13063-021-05199-4 33794971 PMC8015312

[67] Pihkala H Cederström A Sandlund M. Beardslee's preventive family intervention for children of mentally ill parents: a swedish national survey. Int J Ment Health Promot. (2010) 12:29–38. 10.1080/14623730.2010.9721804

[68] Strand J Meyersson N. Parents with psychosis and their children: experiences of Beardslee's intervention. Int J Ment Health Nurs. (2020) 26:908–20. 10.1111/inm.12725 32304272

[69] Strand J Rudolfsson L. A qualitative evaluation of professionals' experiences of conducting Beardslee's family intervention in families with parental psychosis. Int J Ment Health Promot. (2017) 19:289–300. 10.1080/14623730.2017.1345690

[70] Lederer J McHugh M FWA. Newpin - working with parents with mental health problems and their young children. Ment Heal Rev J. (2006) 11:23–7. 10.1108/13619322200600038

[71] Mueller B Fellmann L. Supporting children of parents with mental health problems through professionally assisted lay support–the “godparents” program. Child Youth Serv. (2019) 40:23–42. 10.1080/0145935X.2018.1526071

[72] Brunette MF Richardson F White L Bemis G Eelkema RE. Integrated family treatment for parents with severe psychiatric disabilities. Psychiatr Rehabil J. (2004) 28:177–80. 10.2975/28.2004.177.180 15609444

[73] OurTime. KidsTime Workshop Manual. London: OurTime (2020).

[74] Ford DM. The ‘Kidstime’ Intervention for Children of Parents With Mental Illness: An Exploration of the Experience of the ‘Kidstime’ Workshops and Relevant School-Based Support. Ph.D. thesis. (2019).

[75] Maybery DJ Goodyear M Reupert AE Sheen J Cann W O'Hanlon B. A mixed method evaluation of an intervention for parents with mental illness. Clin Child Psychol Psychiatry. (2019) 24:717–27. 10.1177/1359104518822676 30696254

[76] Maybery DJ Goodyear M Reupert AE Sheen J Cann W Dalziel K. Developing an Australian-first recovery model for parents in Victorian mental health and family services: a study protocol for a randomised controlled trial. BMC Psychiatry. (2017). 17:198. 10.1186/s12888-017-1357-4 28549427 PMC5446721

[77] Cooper V Reupert AE. “Let's talk about children” resource: a parallel mixed method evaluation. Soc Work Ment Health. (2017) 15:47–65. 10.1080/15332985.2016.1170090

[78] Bassett H Lampe J Lloyd C. Living with under-fives: a programme for parents with a mental illness. Br J Occup Ther. (2001) 64:23–8. 10.1177/030802260106400105

[79] Bassett H Lloyd C. At-risk families with mental illness: partnerships in practice. New Zeal J Occup Ther. (2005) 52:31–7.24138021

[80] Kaplan K Solomon P Salzer MS Brusilovskiy E. Assessing an Internet-based parenting intervention for mothers with a serious mental illness: a randomized controlled trial. Psychiatr Rehabil J. (2014) 37:222–31. 10.1037/prj0000080 24978623

[81] van der Ende PC Venderink MM van Busschbach JT. Parenting with success and satisfaction among parents with severe mental illness. Psychiatr Serv. (2010) 61:416. 10.1176/ps.2010.61.4.416 20360284

[82] van der Ende PC. Vulnerable Parenting, A Study on Parents With Mental Health Problems: Strategies and Support. Groningen: Hanze University of Applied Sciences Groningen. (2016).

[83] van der Ende PC van Busschbach JT Nicholson J Korevaar EL van Weeghel J. Parenting and psychiatric rehabilitation: can parents with severe mental illness benefit from a new approach? Psychiatr Rehabil J. (2014) 37:201–8. 10.1037/prj0000067 24866839

[84] Wansink HJ Hosman CMH Janssens JMAM Hoencamp E Willems WJCT. Preventive family service coordination for parents with a mental illness in the Netherlands. Psychiatr Rehabil J. (2014) 37:216–21. 10.1037/prj0000073 24819697

[85] Wansink HJ Janssens JMAM Hoencamp E Middelkoop BJC Hosman CMH. Effects of preventive family service coordination for parents with mental illnesses and their children, a RCT. Fam Syst Heal. (2015) 33:110–9. 10.1037/fsh0000105 25751176

[86] Fritz L-M Domin S Thies A Yang J Stolle M Fricke C. Profitieren psychisch erkrankte Eltern und psychisch belastete Kinder von einer gemeinsamen Eltern-Kind-Behandlung? Kindheit und Entwicklung. (2018) 27:253–67. 10.1026/0942-5403/a00026428686276

[87] McFarland L Fenton A. Unfogging the future: investigating a strengths-based program to build capacity and resilience in parents with mental illness. Adv Ment Heal. (2019) 17:21–32. 10.1080/18387357.2018.1476065

[88] Volkert J Georg A Hauschild S Herpertz SC Neukel C Byrne G. Bindungskompetenzen psychisch kranker Eltern stärken: adaptation und pilottestung des mentalisierungsbasierten leuchtturm-elternprogramms. Prax Kinderpsychol Kinderpsychiatr. (2019) 68:27–42. 10.13109/prkk.2019.68.1.27 30628875

[89] Shor R Kalivatz Z Amir Y Aldor R Lipot M. Therapeutic factors in a group for parents with mental illness. Community Ment Health J. (2015) 51:79–84. 10.1007/s10597-014-9739-2 24962269

[90] Gatsou L Yates S Goodrich N Pearson D. The challenges presented by parental mental illness and the potential of a whole-family intervention to improve outcomes for families. Child Fam Soc Work. (2017) 22:388–97. 10.1111/cfs.12254

[91] Kuschel A Granic M Hahlweg K Hartung D. ≪Nicht von schlechten Eltern!≫ Effekte einer therapieintegrierten Familienintervention. Verhaltenstherapie. (2016) 26:83–91. 10.1159/000446170

[92] Butler J Gregg L Calam R Wittkowski A. Exploring staff implementation of a self-directed parenting intervention for parents with mental health difficulties. Community Ment Health J. (2021) 57:247–61. 10.1007/s10597-020-00642-3 32445074 PMC7835308

[93] Wolfenden LL. Parental Psychosis: Exploring Emotional and Cognitive Processes and the Feasibility of a Parenting Intervention. Ph.D. thesis, University of Manchester (2018).

[94] Müller AD Gjøde ICT Eigil MS Busck H Bonne M Nordentoft M. VIA family - a family-based early intervention versus treatment as usual for familial high-risk children: a study protocol for a randomized clinical trial. Trials. (2019) 20:1–17. 10.1186/s13063-019-3191-0 30736834 PMC6368720

[95] Riemersma I van Santvoort F Van Doesum KT Hosman CM Janssens JMA Van Der Zanden RAP. ‘You are Okay’: effects of a support and educational program for children with mild intellectual disability and their parents with mental health concerns. J Intellect Disabil. (2020). 2020:174462952095376. 10.1177/1744629520953765 32909887

[96] Riemersma I van Santvoort F Janssens JMA Hosman CM van Doesum KT. “You are Okay”: a support and educational program for children with mild intellectual disability and their parents with a mental illness: study protocol of a quasiexperimental design. BMC Psychiatry. (2015) 15:318. 10.1186/s12888-015-0698-0 26702610 PMC4690258

[97] Abel KM Bee P Gega L Gellatly J Kolade A Hunter D. An intervention to improve the quality of life in children of parents with serious mental illness: the Young SMILES feasibility RCT. Health Technol Assess. (2020) 24:1–136. 10.3310/hta24590 33196410 PMC7701992

[98] Gellatly J Bee P Gega L Bower P Hunter D Stewart P. A community-based intervention (Young SMILES) to improve the health-related quality of life of children and young people of parents with serious mental illness: randomised feasibility protocol. Trials. (2018) 19:550. 10.1186/s13063-018-2935-6 30314509 PMC6186077

[99] Sanders MR. Triple P-positive parenting program as a public health approach to strengthening parenting. J Fam Psychol. (2008) 22:506–17. 10.1037/0893-3200.22.3.506 18729665

[100] Phelan R Lee L Howe D Walter G. Parenting and mental illness: a pilot group programme for parents. Aust Psychiatry. (2006) 14:399–402. 10.1080/j.1440-1665.2006.02312.x 17116080

[101] Havighurst SS Harley AE Prior MR. Tuning into kids: an emotion-focused parenting program - initial findings from a community trial. J Community Psychol. (2009) 37:1008–23. 10.1002/jcop.2034525855820

[102] Fadden G Heelis R. The Meriden Family Programme: lessons learned over 10 years. J Ment Heal. (2011) 20:79–88. 10.3109/09638237.2010.492413 20812854

[103] Coates D Phelan R Heap J Howe D. “Being in a group with others who have mental illness makes all the difference”: the views and experiences of parents who attended a mental health parenting program. Child Youth Serv Rev. (2017). 78:104–11. 10.1016/j.childyouth.2017.05.015

[104] Higgins JP Thomas J Chandler J Cumpston M Li T Page MJ. Cochrane Handbook for Systematic Reviews of Interventions. Chichester UK: John Wiley & Sons (2019). 10.1002/9781119536604 PMC10284251 31643080

[105] Hosman CM van Doesum KT van Santvoort F. Prevention of emotional problems and psychiatric risks in children of parents with a mental illness in the Netherlands: II. Intervent. Aust. e-J. Adv Ment Heal. (2009) 8:264–76. 10.5172/jamh.8.3.264

[106] Venkataraman MJ Ackerson B. Parenting among mothers with bipolar disorder: strengths, challenges, and service needs. J Fam Soc Work. (2008) 11:389–408. 10.1080/10522150802441825

[107] Birchwood M Spencer E McGovern D. Schizophrenia: early warning signs. Adv Psychiatr Treat. (2000) 6:93–101. 10.1192/apt.6.2.93

[108] Radley J Barlow J Johns L. Mental health professionals' experiences of working with parents with psychosis and their families : a qualitative study. BMC Health Serv Res. (2021) 1:1–11. 10.1186/s12913-021-06416-1 33906656 PMC8077930

[109] Radley J Barlow J Johns L. The needs and experiences of parents with psychosis: a qualitative interview study (2021). Submitted.

[110] Repper J Carter T. A review of the literature on peer support in mental health services. J Ment Heal. (2011) 20:392–411. 10.3109/09638237.2011.583947 21770786

[111] Nicholson J Valentine A. Key informants specify core elements of peer supports for parents with serious mental illness. Front Psychiatry. (2019) 10:106. 10.3389/fpsyt.2019.00106 30886592 PMC6409303

[112] Nicholson J Reupert AE Grant A Lees R Maybery DJ Mordoch E. The policy context and change for families living with parental mental illness. In: Reupert AE Maybery DJ Joanne N Gopfert M Seeman MV, editors. Parental Psychiatric Disorder Distressed Parents and Their Families. Cambridge: Cambridge University Press (2015). p. 354–64. 10.1017/CBO9781107707559.034

[113] Lauritzen C Reedtz C Rognmo K Nilsen MA Walstad A. Identification of and support for children of mentally ill parents: a 5 year follow-up study of adult mental health services. Front Psychiatry. (2018) 9:507. 10.3389/fpsyt.2018.00507 30386268 PMC6198071

[114] Mehta UM Bhagyavathi HD Kumar CN Thirthalli J Gangadhar BN. Cognitive deconstruction of parenting in schizophrenia: the role of theory of mind. Aust N Z J Psychiatry. (2014) 48:249–58. 10.1177/0004867413500350 23928275

[115] Røhder K Nyström-Hansen M MacBeth A Davidsen KA Gumley A Brennan J. Antenatal caregiving representations among expectant mothers with severe mental illness: a cross-sectional study. J Reprod Infant Psychol. (2019) 37:370–83. 10.1080/02646838.2019.1578868 30767656

[116] Marston N Stavnes K Van Loon LMA Drost LM Maybery DJ Mosek A. A content analysis of Intervention Key Elements and Assessments (IKEA): what's in the black box in the interventions directed to families where a parent has a mental illness? Child Youth Serv. (2016). 37:112–28. 10.1080/0145935X.2016.1104041

[117] Strand J Boström PK Grip K. Parents' descriptions of how their psychosis affects parenting. J Child Fam Stud. (2020) 29:620–31. 10.1007/s10826-019-01605-3

[118] Wansink HJ Drost RMWA Paulus Aggie TG Ruwaard D Hosman CMH. Cost-effectiveness of preventive case management for parents with a mental illness: a randomized controlled trial from three economic perspectives. BMC Health Serv Res. (2016). 16:228. 10.1186/s12913-016-1498-z 27388373 PMC4937554

[119] Kivelkitz L Dirmaier J Harter M. Entscheidungshilfe - ambulante oder stationare behandlungs - moglichkeiten. Psychenet. (2020). Available online at: https://psychenet.de/de/entscheidungshilfen/ambulante-oder-stationaere-behandlungsmoeglichkeiten/das-versorgungssystem-behandlungsmoeglichkeiten/das-versorgungssystem-behandlungsmoeglichkeiten.html (accessed September 28, 2021).

[120] Fekadu W Mihiretu A Craig TKJ Fekadu A. Multidimensional impact of severe mental illness on family members: systematic review. BMJ Open. (2019) 9:1–12. 10.1136/bmjopen-2019-032391 31892656 PMC6955519

[121] Dean K Stevens H Mortensen PB Murray RM Walsh E Pedersen CB. Full spectrum of psychiatric outcomes among offspring with parental history of mental disorder. Arch Gen Psychiatry. (2010) 67:822–9. 10.1001/archgenpsychiatry.2010.86 20679590

[122] Waldmann T Stiawa M Dinc Ü Saglam G Busmann M Daubmann A. Costs of health and social services use in children of parents with mental illness. Child Adolesc Psychiatry Ment Health. (2021) 15:1–11. 10.1186/s13034-021-00360-y 33610177 PMC7897390

