# Supplementary material for: A Scoping Review of Interventions Designed to Support Parents With Mental Illness That Would Be Appropriate for Parents With Psychosis
Source: Front Psychiatry. 2022 Jan 27;12:787166. doi: 10.3389/fpsyt.2021.787166 (PMC8828543; doi:10.3389/fpsyt.2021.787166)
Supplement: Supplementary file 2 [file Table_2.DOCX]

# Supplementary file 2

Table 5 - All 110 reports found in search, and their corresponding interventions

| Intervention | No. of reports | Primary report(s) | Other report(s) |
| --- | --- | --- | --- |
| Child Resilience Programme | 1 | (Gutjahr 2007) |  |
| Child Talks+ | 8 | (Van Doesum, Lauritzen, and Reedtz 2020)  (Reedtz et al. 2019) | (Reedtz, Lauritzen, and Van Doesum 2012; Lauritzen 2014; Lauritzen et al. 2014, 2018; Van Doesum et al. 2019; van Doesum and Hosman 2009) |
| Family Options | 13 | (Nicholson et al. 2009, 2016) | (Biebel, Nicholson, and Woolsey 2014; Nicholson 2007; Hinden and Mikula 2008; Hinden et al. 2009; Dvir 2012; Hinden and Wilder 2008; Biebel, Nicholson, and Wolf 2015; Biebel et al. 2016; Nicholson and Valentine 2018, 2019; Nicholson, Wolf, and Biebel 2015) |
| Family Talk | 3 | (Pihkala, Cederström, and Sandlund 2010) | (Pihkala, Sandlund, and Cederström 2012a, 2012b) |
|  | 3 | (Furlong et al. 2021) | (Mulligan, Furlong, and McGilloway 2019), ISRCTN13365858 |
|  | 2 | (Strand and Rudolfsson 2017; Strand and Meyersson 2020) |  |
|  | 1 | (Christiansen et al. 2015) |  |
| FWA Newpin service | 1 | (Lederer and Mchugh 2006) |  |
| Godparents programme | 1 | (Mueller and Fellmann 2019) |  |
| Integrated Family Treatment | 1 | (Brunette et al. 2004) |  |
| Invisible Children's Project | 1 | (Hinden et al. 2005) |  |
| KidsTime | 8 | (OurTime 2020; Ford 2019) | (Cooklin et al. 2012; Cooklin 2013; Wolpert et al. 2015; Cooklin and Barnes 2020; Cardenas 2020; Spierling 2020) |
| KopOpOuders | 1 | (Van Der Zanden et al. 2010) |  |
| Effective Child and Family Program EC&F | 3 | (Solantaus, Reupert, and Maybery 2015; Solantaus and Toikka 2006) | (Toikka and Solantaus 2006) |
| Let's Talk about children | 12 | (Maybery et al. 2017, 2019), ACTRN12616000460404 | (Powell, Ross, and Jessop 2015; Tchernegovski, Reupert, and Maybery 2015; Afzelius, Plantin, and Östman 2018; Karibi and Arblaster 2019; Allchin, O’Hanlon, Weimand, Boyer, et al. 2020; Allchin, Goodyear, O’Hanlon, and Weimand 2020; Allchin, O’Hanlon, Weimand, and Goodyear 2020; Allchin, Weimand, O’Hanlon, and Goodyear 2020; Niemelä et al. 2019) |
| Let's Talk about Children booklet | 1 | (Cooper and Reupert 2017) |  |
| Living with Under Fives | 3 | (Bassett, Lampe, and Lloyd 2001; Bassett and Lloyd 2005) | (Bassett, Lampe, and Lloyd 1999) |
| Parenting with Success and Satisfaction workbooks | 4 | (van der Ende, Venderink, and van Busschbach 2010; van der Ende et al. 2014; van der Ende 2016) | (van der Ende et al. 2017) |
| Preventive basic care management (PBCM) | 3 | (Wansink et al. 2014, 2015) | (Wansink et al. 2016) |
| Think Family Whole Family Programme | 4 | (Gatsou et al. 2017) | (Yates and Gatsou 2017, 2021; Gatsou et al. 2015) |
| Triple P - Every Parent’s Self-Help Workbook | 1 | (Butler et al. 2021) |  |
| Triple P Self-Help Workbook | 3 | (Wolfenden 2018) | NCT02622048, NCT02199704 |
| Triple P + CBT | 2 | (Stracke et al. 2019) | (Christiansen et al. 2019) |
| Triple P + mental health components | 3 | (Phelan et al. 2012) | (Phelan et al. 2006; Coates et al. 2017) |
| Triple P | 1 | (Kuschel et al. 2016) |  |
| Tuning into Kids | 1 | (Isobel, Meehan, and Pretty 2016) |  |
| Child and Family Inclusive Programme | 2 | (Cowling and Garrett 2009, 2012) |  |
| Parenting internet intervention | 3 | (Kaplan et al. 2014) | (Kaplan 2013; O’Shea et al. 2019) |
| Counselling and support service (unnamed) | 1 | (Becker et al. 2009) |  |
| Therapeutic group (unnamed) | 1 | (Shor et al. 2015) |  |
| Strengths based parenting programme (unnamed) | 1 | (McFarland and Fenton 2019) |  |
| VIA Family | 3 | (Müller et al. 2019) | (Thorup et al. 2018), NCT03497663 |
| You are Okay | 3 | (Riemersma et al. 2015, 2020) | (Riemersma, van Santvoort, and Varmaes 2014) |
| Young SMILES | 4 | (Abel et al. 2020; Gellatly et al. 2018) | (Gellatly et al. 2019), ISRCTN36865046 |
| CHIMPS intervention | 4 | (Wiegand-Grefe et al. 2021) | NCT02308462, NCT04369625 (Waldmann et al. 2021) |
| The Lighthouse (Leuchtturm) Parenting Programme | 1 | (Volkert et al. 2019) |  |
| SEEK | 1 | (Fritz et al. 2018) |  |
| BROSH Program | 1 | (Oppenheim-Weller, Shtark, and Aldor 2021) |  |

Abel, Kathryn M, Penny Bee, Lina Gega, Judith Gellatly, Adekeye Kolade, Diane Hunter, Craig Callender, et al. 2020. “An Intervention to Improve the Quality of Life in Children of Parents with Serious Mental Illness: The Young SMILES Feasibility RCT.” *Health Technology Assessment (Winchester, England)* 24 (59): 1–136. https://doi.org/10.3310/hta24590.

Afzelius, M., L. Plantin, and M. Östman. 2018. “Families Living with Parental Mental Illness and Their Experiences of Family Interventions.” *Journal of Psychiatric and Mental Health Nursing* 25 (2): 69–77. https://doi.org/10.1111/jpm.12433.

Allchin, Becca, Melinda Goodyear, Brendan O’Hanlon, and Bente M. Weimand. 2020. “Leadership Perspectives on Key Elements Influencing Implementing a Family-Focused Intervention in Mental Health Services.” *Journal of Psychiatric and Mental Health Nursing* 27 (5): 616–27. https://doi.org/10.1111/jpm.12615.

Allchin, Becca, Brendan O’Hanlon, Bente M. Weimand, Fran Boyer, Georgia Cripps, Lisa Gill, Brooke Paisley, Sian Pietsch, Brad Wynne, and Melinda Goodyear. 2020. “An Explanatory Model of Factors Enabling Sustainability of Let’s Talk in an Adult Mental Health Service: A Participatory Case Study.” *International Journal of Mental Health Systems* 14 (1): 1–16. https://doi.org/10.1186/s13033-020-00380-9.

Allchin, Becca, Brendan O’Hanlon, Bente M. Weimand, and Melinda Goodyear. 2020. “Practitioners’ Application of Let’s Talk about Children Intervention in Adult Mental Health Services.” *International Journal of Mental Health Nursing* 29 (5): 899–907. https://doi.org/10.1111/inm.12724.

Allchin, Becca, Bente M. Weimand, Brendan O’Hanlon, and Melinda Goodyear. 2020. “Continued Capacity: Factors of Importance for Organizations to Support Continued Let’s Talk Practice – a Mixed-Methods Study.” *International Journal of Mental Health Nursing* 29 (6): 1131–43. https://doi.org/10.1111/inm.12754.

Bassett, Hazel, Jill Lampe, and Chris Lloyd. 1999. “Meeting the Needs of Parents with Mental Illness.” *British Journal of Therapy and Rehabilitation* 60 (4): 176–80.

———. 2001. “Living with Under-Fives: A Programme for Parents with a Mental Illness.” *British Journal of Occupational Therapy* 64 (1): 23–28. https://doi.org/10.1177/030802260106400105.

Bassett, Hazel, and Chris Lloyd. 2005. “At-Risk Families with Mental Illness: Partnerships in Practice.” *New Zealand Journal of Occupational Therapy* 52 (2): 31–37. https://ezproxy.lib.uconn.edu/login?url=https://search.ebscohost.com/login.aspx?direct=true&db=rzh&AN=106547009&site=ehost-live.

Becker, Thomas, S Kilian, R Killian, C Lahmeyer, and Silvia Krumm. 2009. “Family Needs, Children and Parenthood in People with Mental Illness.” *European Psychiatry* 24 (S1): 1–1. https://doi.org/10.1016/s0924-9338(09)70281-0.

Biebel, Kathleen, Joanne Nicholson, and Toni Wolf. 2015. “Shifting the Intervention Paradigm from Individuals to Families Living with Parental Mental Illness.” In *Parental Psychiatric Disorder. Distressed Parents and Their Families*, edited by Andrea Reupert, Darryl J Maybery, J Nicholson, Michael Gopfert, and Mary V Seeman, Third edit, 343–53. Cambridge: Cambridge University Press.

Biebel, Kathleen, Joanne Nicholson, and Katherine Woolsey. 2014. “Implementing an Intervention for Parents with Mental Illness: Building Workforce Capacity.” *Psychiatric Rehabilitation Journal*, February 10, 2014. http://psycnet.apa.org/journals/prj/37/3/209.html?uid=2014-05050-001.

Biebel, Kathleen, Joanne Nicholson, Katherine Woolsey, and Toni Wolf. 2016. “Shifting an Agency’s Paradigm: Creating the Capacity to Intervene with Parents with Mental Illness.” *American Journal of Psychiatric Rehabilitation* 19 (4): 315–38. https://doi.org/10.1080/15487768.2016.1231641.

Brunette, Mary F., Fred Richardson, Laurie White, Gillian Bemis, and Rachel E. Eelkema. 2004. “Integrated Family Treatment for Parents with Severe Psychiatric Disabilities.” *Psychiatric Rehabilitation Journal* 28 (2): 177–80. https://doi.org/10.2975/28.2004.177.180.

Butler, Jennifer, Lynsey Gregg, Rachel Calam, and Anja Wittkowski. 2021. “Exploring Staff Implementation of a Self-Directed Parenting Intervention for Parents with Mental Health Difficulties.” *Community Mental Health Journal* 57 (2): 247–61. https://doi.org/10.1007/s10597-020-00642-3.

Cardenas, Miguel. 2020. “Kidstime Experience in Spain.” In *Building Children’s Resilience in the Face of Parental Mental Illness*, edited by Alan Cooklin and Gill Gorell Barnes, 211–13. Routledge.

Christiansen, Hanna, Jana Anding, Bastian Schrott, and Bernd Röhrle. 2015. “Children of Mentally Ill Parents-A Pilot Study of a Group Intervention Program.” *Frontiers in Psychology* 6 (OCT). https://doi.org/10.3389/fpsyg.2015.01494.

Christiansen, Hanna, Corinna Reck, Anna Lena Zietlow, Kathleen Otto, Ricarda Steinmayr, Linda Wirthwein, Sarah Weigelt, et al. 2019. “Children of Mentally III Parents at Risk Evaluation (COMPARE): Design and Methods of a Randomized Controlled Multicenter Study—Part I.” *Frontiers in Psychiatry* 10 (MAR). https://doi.org/10.3389/fpsyt.2019.00128.

Coates, Dominiek, Ruth Phelan, Joanna Heap, and Deborah Howe. 2017. “‘Being in a Group with Others Who Have Mental Illness Makes All the Difference’: The Views and Experiences of Parents Who Attended a Mental Health Parenting Program.” *Children and Youth Services Review* 78 (January): 104–11. https://doi.org/10.1016/j.childyouth.2017.05.015.

Cooklin, Alan. 2013. “Promoting Children’s Resilience to Parental Mental Illness: Engaging the Child’s Thinking.” *Advances in Psychiatric Treatment* 19 (03): 229–40. https://doi.org/10.1192/apt.bp.111.009050.

Cooklin, Alan, and Gill Gorell Barnes. 2020. *Building Children’s Resilience in the Face of Parental Mental Illness*. *Routledge*. https://doi.org/10.1080/18387357.2021.1876521.

Cooklin, Alan, Peter Bishop, Deni Francis, Leonard Fagin, and Eia Asen. 2012. *The Kidstime Workshops a Multi-Family Social Intervention for the Effects of Parental Mental Illness Manual*. https://www.annafreud.org/media/3191/book_kidstime-manual-final-version.pdf.

Cooper, Vicki, and Andrea E Reupert. 2017. “‘Let’s Talk About Children’ Resource: A Parallel Mixed Method Evaluation.” *Social Work in Mental Health* 15 (1): 47–65. https://doi.org/10.1080/15332985.2016.1170090.

Cowling, Vicki, and Matthew Garrett. 2009. “Child and Family Inclusive Practice: A Pilot Program in a Community Adult Mental Health Service.” *Australasian Psychiatry* 17 (4): 279–82. https://doi.org/10.1080/10398560902840232.

———. 2012. “A Child-Inclusive Family Intervention in a Community Adult Mental Health Service.” *Australian and New Zealand Journal of Family Therapy* 33 (2): 101–13. https://doi.org/10.1017/aft.2012.13.

Doesum, Karin TM van, and Clemens MH Hosman. 2009. “Prevention of Emotional Problems and Psychiatric Risks in Children of Parents with a Mental Illness in the Netherlands: II. Interventions.” *Australian E-Journal for the Advancement of Mental Health* 8 (3): 264–76. https://doi.org/10.5172/jamh.8.3.264.

Doesum, Karin TM Van, Camilla Lauritzen, and Charlotte Reedtz. 2020. *Child Talks+ Manual*.

Doesum, Karin TM Van, Teresa Maia, Catarina Pereira, Monica Loureiro, Joana Marau, Lurdes Toscano, Camilla Lauritzen, and Charlotte Reedtz. 2019. “The Impact of the ‘SEMENTE’ Program on the Family-Focused Practice of Mental Health Professionals in Portugal.” *Frontiers in Psychiatry* 10 (MAY). https://doi.org/10.3389/fpsyt.2019.00305.

Dvir, Yael. 2012. “Parenting and Mental Illness: A Group for Mothers.” *Frontiers in Psychiatry* 3 (July): 1–2. https://doi.org/10.3389/fpsyt.2012.00071.

Ende, Peter C van der. 2016. *Vulnerable Parenting, a Study on Parents with Mental Health Problems: Strategies and Support*. Groningen: Hanze University of Applied Sciences Groningen.

Ende, Peter C van der, Jooske T van Busschbach, Joanne Nicholson, Eliza L Korevaar, and Jaap van Weeghel. 2014. “Parenting and Psychiatric Rehabilitation: Can Parents with Severe Mental Illness Benefit from a New Approach?” *Psychiatric Rehabilitation Journal* 37 (3): 201–8. https://doi.org/10.1037/prj0000067.

Ende, Peter C van der, Lies Korevaar, Jooske T. van Busschbach, and Jaap van Weeghel. 2017. “Professionals’ Opinions on Support for People with Chronic Illness in Their Roles as Parents in Mental or in General Health Care.” *American Journal of Psychiatric Rehabilitation* 20 (1): 74–86. https://doi.org/10.1080/15487768.2016.1267048.

Ende, Peter C van der, Marrie M Venderink, and Jooske T van Busschbach. 2010. “Parenting With Success and Satisfaction Among Parents With Severe Mental Illness.” *Psychiatric Services* 61 (4): 416. https://doi.org/10.1176/ps.2010.61.4.416.

Ford, Danielle Marie. 2019. “The ‘Kidstime’ Intervention for Children of Parents with Mental Illness: An Exploration of the Experience of the ‘Kidstime’ Workshops and Relevant School-Based Support.” *PhD Thesis*.

Fritz, Lisa-Marina, Sabine Domin, Annekatrin Thies, Julia Yang, Martin Stolle, Christian Fricke, and Franz Petermann. 2018. “Profitieren Psychisch Erkrankte Eltern Und Psychisch Belastete Kinder von Einer Gemeinsamen Eltern-Kind-Behandlung?” *Kindheit Und Entwicklung* 27 (4): 253–67. https://doi.org/10.1026/0942-5403/a000264.

Furlong, Mairead, Sinead McGilloway, Christine Mulligan, Colm McGuinness, and Nuala Whelan. 2021. “Family Talk versus Usual Services in Improving Child and Family Psychosocial Functioning in Families with Parental Mental Illness (PRIMERA—Promoting Research and Innovation in Mental HEalth SeRvices for FAmilies and Children): Study Protocol for a Randomi.” *Trials* 22 (1): 1–18. https://doi.org/10.1186/s13063-021-05199-4.

Gatsou, Lina, Scott Yates, G Fadden, N Goodrich, and Dan Pearson. 2015. “Think Family - Whole Family Programme: Improving the Outcomes for Families Affected by Parental Mental Illness.” In *European Child and Adolescent Psychiatry*, S33-33. New York: Springer.

Gatsou, Lina, Scott Yates, Nigel Goodrich, and Dan Pearson. 2017. “The Challenges Presented by Parental Mental Illness and the Potential of a Whole-Family Intervention to Improve Outcomes for Families.” *Child and Family Social Work* 22 (1): 388–97. https://doi.org/10.1111/cfs.12254.

Gellatly, Judith, Penny Bee, Lina Gega, Peter Bower, Diane Hunter, Paul Stewart, Nicky Stanley, et al. 2018. “A Community-Based Intervention (Young SMILES) to Improve the Health-Related Quality of Life of Children and Young People of Parents with Serious Mental Illness: Randomised Feasibility Protocol.” *Trials* 19 (October). https://doi.org/10.1186/s13063-018-2935-6.

Gellatly, Judith, Penny Bee, Adekeye Kolade, Diane Hunter, Lina Gega, Craig Callender, Holly Hope, and Kathryn M Abel. 2019. “Developing an Intervention to Improve the Health Related Quality of Life in Children and Young People with Serious Parental Mental Illness.” *Frontiers in Psychiatry* 10 (APR): 1–12. https://doi.org/10.3389/fpsyt.2019.00155.

Gutjahr, Angela. 2007. “Child Resilience Program an Intervention for Children of Chronically Mentally Ill Parents.” *PhD Thesis*.

Hinden, Beth R, Kathleen Biebel, Joanne Nicholson, and Liz Mehnert. 2005. “The Invisible Children’ s Project: Key Ingredients of an Intervention for Parents with Mental Illness.” *The Journal of Behavioral Health Services & Research* 32 (4): 393–408.

Hinden, Beth R, and J Mikula. 2008. “Implementation Challenges in Wrapping Interventions around Families with Parental Mental Illness.” In *Systems of Care*, 271. https://doi.org/10.1161/circulationaha.108.790170.

Hinden, Beth R, and Chip Wilder. 2008. “Family Options: Supporting Parents with Mental Illness and Their Children.” *Focal Point* 22 (2): 7–9.

Hinden, Beth R, Toni Wolf, Kathleen Biebel, and Joanne Nicholson. 2009. “Supporting Clubhouse Members in Their Role as Parents: Necessary Conditions for Policy and Practice Initiatives.” *Psychiatric Rehabilitation Journal* 33 (2): 98–105. https://doi.org/10.2975/33.2.2009.98.105.

Isobel, Sophie, Felicity Meehan, and Danielle Pretty. 2016. “An Emotional Awareness Based Parenting Group for Parents with Mental Illness: A Mixed Methods Feasibility Study of Community Mental Health Nurse Facilitation.” *Archives of Psychiatric Nursing* 30 (1): 35–40. https://doi.org/10.1016/j.apnu.2015.10.007.

Kaplan, Katy. 2013. “Assessing the Impact of an Internet-Based Parenting Intervention for Mothers with Psychiatric Disabilities: A Randomised Controlled Trial.” *PhD Thesis*.

Kaplan, Katy, Phyllis Solomon, Mark S. Salzer, and Eugene Brusilovskiy. 2014. “Assessing an Internet-Based Parenting Intervention for Mothers with a Serious Mental Illness: A Randomized Controlled Trial.” *Psychiatric Rehabilitation Journal* 37 (3): 222–31. https://doi.org/10.1037/prj0000080.

Karibi, Hanien, and Karen Arblaster. 2019. “Clinician Experiences of ‘Let’s Talk about Children’ Training and Implementation to Support Families Affected by Parental Mental Illness.” *Journal of Mental Health Training, Education and Practice* 14 (4): 201–11. https://doi.org/10.1108/JMHTEP-08-2018-0044.

Kuschel, Annett, Mara Granic, Kurt Hahlweg, and Doreen Hartung. 2016. “«Nicht von Schlechten Eltern!» Effekte Einer Therapieintegrierten Familienintervention.” *Verhaltenstherapie* 26 (2): 83–91. https://doi.org/10.1159/000446170.

Lauritzen, Camilla. 2014. “Implementing Interventions in Adult Mental Health Services to Identify and Support Children of Mentally Ill Parents.” *PhD Thesis*. https://munin.uit.no/handle/10037/6729.

Lauritzen, Camilla, Charlotte Reedtz, Karin Tm Van Doesum, and Monica Martinussen. 2014. “Implementing New Routines in Adult Mental Health Care to Identify and Support Children of Mentally Ill Parents.” *BMC Health Services Research* 14. https://doi.org/10.1186/1472-6963-14-58.

Lauritzen, Camilla, Charlotte Reedtz, Kamilla Rognmo, Miriam A. Nilsen, and Anja Walstad. 2018. “Identification of and Support for Children of Mentally Ill Parents: A 5 Year Follow-up Study of Adult Mental Health Services.” *Frontiers in Psychiatry* 9 (507): 1–10. https://doi.org/10.3389/fpsyt.2018.00507.

Lederer, Jacqui, and Marian Mchugh. 2006. “FWA Newpin - Working With Parents With Mental Health Problems And Their Young Children.” *Mental Health Review Journal* 11 (4): 23–27. https://doi.org/10.1108/13619322200600038.

Maybery, Darryl J, Melinda Goodyear, Andrea E Reupert, Jade Sheen, Warren Cann, Kim Dalziel, Phillip Tchernegovski, Brendan O’Hanlon, and Henry von Doussa. 2017. “Developing an Australian-First Recovery Model for Parents in Victorian Mental Health and Family Services: A Study Protocol for a Randomised Controlled Trial.” *BMC Psychiatry* 17 (1). https://doi.org/10.1186/s12888-017-1357-4.

Maybery, Darryl J, Melinda Goodyear, Andrea E Reupert, Jade Sheen, Warren Cann, Brendan O’Hanlon, and Rose Cuff. 2019. “A Mixed Method Evaluation of an Intervention for Parents with Mental Illness.” *Clinical Child Psychology and Psychiatry*. https://doi.org/10.1177/1359104518822676.

McFarland, Laura, and Angela Fenton. 2019. “Unfogging the Future: Investigating a Strengths-Based Program to Build Capacity and Resilience in Parents with Mental Illness.” *Advances in Mental Health* 17 (1): 21–32. https://doi.org/10.1080/18387357.2018.1476065.

Mueller, Brigitte, and Lukas Fellmann. 2019. “Supporting Children of Parents with Mental Health Problems through Professionally Assisted Lay Support–the ‘Godparents’ Program.” *Child and Youth Services* 40 (1): 23–42. https://doi.org/10.1080/0145935X.2018.1526071.

Müller, Anne D., Ida C.T. Gjøde, Mette S. Eigil, Helle Busck, Merete Bonne, Merete Nordentoft, and Anne A E Thorup. 2019. “VIA Family - A Family-Based Early Intervention versus Treatment as Usual for Familial High-Risk Children: A Study Protocol for a Randomized Clinical Trial.” *Trials* 20 (1): 1–17. https://doi.org/10.1186/s13063-019-3191-0.

Mulligan, Christine, Mairead Furlong, and Sinéad McGilloway. 2019. “Promoting and Implementing Family-Focused Interventions for Families with Parental Mental Illness: Scoping and Installation.” *Advances in Mental Health* 0 (0): 1–15. https://doi.org/10.1080/18387357.2019.1614466.

Nicholson, Joanne. 2007. “Helping Parents with Mental Illness.” *Behavioral Healthcare* 27 (5): 32–33.

Nicholson, Joanne, Karen Albert, Bernice Gershenson, Valerie Williams, and Kathleen Biebel. 2009. “Family Options for Parents with Mental Illnesses: A Developmental, Mixed Methods Pilot Study.” *Psychiatric Rehabilitation Journal* 33 (2): 106–14. https://doi.org/10.2975/33.2.2009.106.114.

———. 2016. “Developing Family Options: Outcomes for Mothers with Severe Mental Illness at Twelve Months of Participation.” *American Journal of Psychiatric Rehabilitation* 19 (4): 353–69. https://doi.org/10.1080/15487768.2016.1231639.

Nicholson, Joanne, and Anne Valentine. 2018. “Defining ‘Peerness’: Developing Peer Supports for Parents with Mental Illnesses.” *Psychiatric Rehabilitation Journal* 41 (2): 157–59. https://doi.org/10.1037/prj0000301.

———. 2019. “Key Informants Specify Core Elements of Peer Supports for Parents With Serious Mental Illness.” *Frontiers in Psychiatry* 10 (March). https://doi.org/10.3389/fpsyt.2019.00106.

Nicholson, Joanne, Toni Wolf, and Kathleen Biebel. 2015. “Creating Positive Parenting Experiences: Family Options.” In *Parental Psychiatric Disorder. Distressed Parents and Their Families*, edited by Andrea E Reupert, Darryl J Maybery, Joanne Nicholson, Michael Gopfert, and Mary V Seeman, 266–76. Cambridge: Cambridge University Press.

Niemelä, Mika, Hannu Kallunki, Jaana Jokinen, Sami Räsänen, Birkitta Ala-Aho, Helinä Hakko, Tiina Ristikari, and Tytti Solantaus. 2019. “Collective Impact on Prevention: Let’s Talk about Children Service Model and Decrease in Referrals to Child Protection Services.” *Frontiers in Psychiatry* 10 (FEB). https://doi.org/10.3389/fpsyt.2019.00064.

O’Shea, Amber, Katy Kaplan, Phyllis Solomon, and Mark S. Salzer. 2019. “Randomized Controlled Trial of an Internet-Based Educational Intervention for Mothers with Mental Illnesses: An 18-Month Follow-Up.” *Psychiatric Services* 70 (8): 732–35. https://doi.org/10.1176/appi.ps.201800391.

Oppenheim-Weller, Shani, Tammi Shtark, and Roy Aldor. 2021. “Families with Parental Mental Illness: Studying a Home-Based Intervention Program.” *Child and Family Social Work* 26: 617–28. https://doi.org/10.1111/cfs.12843.

OurTime. 2020. “KidsTime Workshop Manual.”

Phelan, Ruth, Deborah J. Howe, Emma L. Cashman, and Samantha H. Batchelor. 2012. “Enhancing Parenting Skills for Parents with Mental Illness: The Mental Health Positive Parenting Program.” *The Medical Journal of Australia* 199 (3): S30–33. https://doi.org/10.5694/mjao11.11181.

Phelan, Ruth, Lana Lee, Deb Howe, and Garry Walter. 2006. “Parenting and Mental Illness: A Pilot Group Programme for Parents.” *Australasian Psychiatry* 14 (4): 399–402. https://doi.org/10.1111/j.1440-1665.2006.02312.x.

Pihkala, Heljä, Anita Cederström, and Mikael Sandlund. 2010. “Beardslee’s Preventive Family Intervention for Children of Mentally Ill Parents: A Swedish National Survey.” *International Journal of Mental Health Promotion* 12 (1): 29–38. https://doi.org/10.1080/14623730.2010.9721804.

Pihkala, Heljä, Mikael Sandlund, and Anita Cederström. 2012a. “Children in Beardslee’s Family Intervention: Relieved by Understanding of Parental Mental Illness.” *International Journal of Social Psychiatry* 58 (6): 623–28. https://doi.org/10.1177/0020764011419055.

———. 2012b. “Initiating Communication about Parental Mental Illness in Families: An Issue of Confidence and Security.” *International Journal of Social Psychiatry* 58 (3): 258–65. https://doi.org/10.1177/0020764010392088.

Powell, J, D Ross, and Mary Jessop. 2015. “Engaging Families and Carers to Support Recovery.” *Australian & New Zealand Journal of Psychiatry* 49 (1_suppl): 1–122. https://doi.org/10.1177/0004867415578344.

Reedtz, Charlotte, Karin TM van Doesum, Giulia Signorini, Camilla Lauritzen, Therese van Amelsvoort, Floor van Santvoort, Allan H. Young, et al. 2019. “Promotion of Wellbeing for Children of Parents With Mental Illness: A Model Protocol for Research and Intervention.” *Frontiers in Psychiatry* 10. https://doi.org/10.3389/fpsyt.2019.00606.

Reedtz, Charlotte, Camilla Lauritzen, and Karin TM Van Doesum. 2012. “Evaluating Workforce Developments to Support Children of Mentally Ill Parents: Implementing New Interventions in the Adult Mental Healthcare in Northern Norway.” *BMJ Open* 2 (3): 1–6. https://doi.org/10.1136/bmjopen-2011-000709.

Riemersma, Ivon, Floor van Santvoort, Karin T.M Van Doesum, Clemens M.H Hosman, Jan M.A.M Janssens, Rianne A.P. Van Der Zanden, and Roy Otten. 2020. “‘You Are Okay’: Effects of a Support and Educational Program for Children with Mild Intellectual Disability and Their Parents with Mental Health Concerns.” *Journal of Intellectual Disabilities*, 174462952095376. https://doi.org/10.1177/1744629520953765.

Riemersma, Ivon, Floor van Santvoort, Jan M.A.M Janssens, Clemens M.H Hosman, and Karin T.M van Doesum. 2015. “‘You Are Okay’: A Support and Educational Program for Children with Mild Intellectual Disability and Their Parents with a Mental Illness: Study Protocol of a Quasiexperimental Design.” *BMC Psychiatry* 15 (1): 1–9. https://doi.org/10.1186/s12888-015-0698-0.

Riemersma, Ivon, Floor van Santvoort, and I Varmaes. 2014. “Development of an Intervention for Children with a Mild Intellectual Disability of Parents with a Mental Illness (COPMI).” *British Institute of Learning Disabilities.*, 573–84.

Shor, Ron, Zvi Kalivatz, Yael Amir, Roy Aldor, and Marc Lipot. 2015. “Therapeutic Factors in a Group for Parents with Mental Illness.” *Community Mental Health Journal* 51 (1): 79–84. https://doi.org/10.1007/s10597-014-9739-2.

Solantaus, Tytti, Andrea E Reupert, and Darryl J Maybery. 2015. “Working with Parents Who Have a Psychiatric Disorder.” In *Parental Psychiatric Disorder. Distressed Parents and Their Families*, edited by Andrea Reupert, Daryl Maybery, Joanne Nicholson, Michael Gopfert, and Mary V Seeman, Third edit, 238–47. Cambridge: Cambridge University Press.

Solantaus, Tytti, and Sini Toikka. 2006. “The Effective Family Programme: Preventative Services for the Children of Mentally Ill Parents in Finland.” *International Journal of Mental Health Promotion* 8 (3): 37–44.

Spierling, Klaus Henner. 2020. “London Calling - Experiences with the Kidstime Model in Germany.” In *Building Children’s Resilience in the Face of Parental Mental Illness*, edited by Alan Cooklin and Gill Gorell Barnes, 205–10. Routledge.

Stracke, Markus, Kristin Gilbert, Meinhard Kieser, Christina Klose, Johannes Krisam, David D. Ebert, Claudia Buntrock, and Hanna Christiansen. 2019. “CoMPARE Family (Children of Mentally Ill Parents at Risk Evaluation): A Study Protocol for a Preventive Intervention for Children of Mentally Ill Parents (Triple P, Evidence-Based Program That Enhances Parentings Skills, in Addition to Gold-Standard CBT W.” *Frontiers in Psychiatry* 10 (FEB): 1–14. https://doi.org/10.3389/fpsyt.2019.00054.

Strand, Jennifer, and Niklas Meyersson. 2020. “Parents with Psychosis and Their Children: Experiences of Beardslee’s Intervention.” *International Journal of Mental Health Nursing* 26 (5): 908–20. https://doi.org/10.1111/inm.12725.

Strand, Jennifer, and Lisa Rudolfsson. 2017. “A Qualitative Evaluation of Professionals’ Experiences of Conducting Beardslee’s Family Intervention in Families with Parental Psychosis.” *International Journal of Mental Health Promotion* 19 (5): 289–300. https://doi.org/10.1080/14623730.2017.1345690.

Tchernegovski, Phillip, Andrea E Reupert, and Darryl J Maybery. 2015. “‘Let’s Talk about Children’: A Pilot Evaluation of an e-Learning Resource for Mental Health Clinicians.” *Clinical Psychologist* 19 (1): 49–58. https://doi.org/10.1111/cp.12050.

Thorup, Anne A E, Ida Gjode, Anne Dorotee Muller, Mette Signe Eigil, Helle Busck, and Merete Bonne. 2018. “VIA Family – an Integrated, Specialized and Family Based Intervention for Children Born to Parents with Severe Mental Illness.” *Early Intervention in Psychiatry* 12 (October): 104–232. https://doi.org/10.1111/eip.12724.

Toikka, Sini, and Tytti Solantaus. 2006. “The Effective Family Programme II: Clinicians’ Experiences of Training in Promotive and Preventative Child Mental Health Methods.” *International Journal of Mental Health Promotion* 8 (4): 4–10. https://doi.org/10.1080/14623730.2006.9721746.

Volkert, Jana, Anna Georg, Sophie Hauschild, Sabine C. Herpertz, Corinne Neukel, Gerry Byrne, and Svenja Taubner. 2019. “Bindungskompetenzen Psychisch Kranker Eltern Stärken: Adaptation Und Pilottestung Des Mentalisierungsbasierten Leuchtturm-Elternprogramms.” *Praxis Der Kinderpsychologie Und Kinderpsychiatrie* 68 (1): 27–42. https://doi.org/10.13109/prkk.2019.68.1.27.

Waldmann, Tamara, Maja Stiawa, Ümügülsüm Dinc, Gülsah Saglam, Mareike Busmann, Anne Daubmann, Bonnie Adema, Karl Wegscheider, Silke Wiegand-Grefe, and Reinhold Kilian. 2021. “Costs of Health and Social Services Use in Children of Parents with Mental Illness.” *Child and Adolescent Psychiatry and Mental Health* 15 (1): 1–11. https://doi.org/10.1186/s13034-021-00360-y.

Wansink, Henny J, Ruben M. W. A Drost, G Paulus, Aggie T, Dirk Ruwaard, Clemens M. H Hosman, Jan M. A. M Janssens, and Silvia M. A. A Evers. 2016. “Cost-Effectiveness of Preventive Case Management for Parents with a Mental Illness: A Randomized Controlled Trial from Three Economic Perspectives.” *BMC Health Services Research* 16 (1). https://doi.org/10.1186/s12913-016-1498-z.

Wansink, Henny J, Clemens M.H. Hosman, Jan M.A.M. Janssens, Erik Hoencamp, and Willemien J.C.T. Willems. 2014. “Preventive Family Service Coordination for Parents with a Mental Illness in the Netherlands.” *Psychiatric Rehabilitation Journal* 37 (3): 216–21. https://doi.org/10.1037/prj0000073.

Wansink, Henny J, Jan M.A.M. Janssens, Erik Hoencamp, Barend J.C. Middelkoop, and Clemens M.H. Hosman. 2015. “Effects of Preventive Family Service Coordination for Parents with Mental Illnesses and Their Children, a RCT.” *Families, Systems and Health* 33 (2): 110–19. https://doi.org/10.1037/fsh0000105.

Wiegand-Grefe, Silke, Bonnie Filter, Mareike Busmann, Reinhold Kilian, Klaus Thomas Kronmüller, Martin Lambert, Christine Norra, et al. 2021. “Evaluation of a Family-Based Intervention Program for Children of Mentally Ill Parents: Study Protocol for a Randomized Controlled Multicenter Trial.” *Frontiers in Psychiatry* 11 (January): 1–13. https://doi.org/10.3389/fpsyt.2020.561790.

Wolfenden, Lauren L. 2018. “Parental Psychosis: Exploring Emotional and Cognitive Processes and the Feasibility of a Parenting Intervention.” *PhD Thesis*.

Wolpert, Miranda, Jasmine Hoffman, Amelia Martin, Leonard Fagin, and Alan Cooklin. 2015. “An Exploration of the Experience of Attending the Kidstime Programme for Children with Parents with Enduring Mental Health Issues: Parents’ and Young People’s Views.” *Clinical Child Psychology and Psychiatry* 20 (3). https://doi.org/10.1177/1359104514520759.

Yates, Scott, and Lina Gatsou. 2017. “Enhancing Family Communication in Families Where a Parent Has a Mental Illness.” *Journal of Parent & Family Mental Health* 2 (3): 1–3.

———. 2021. “Undertaking Family-Focused Interventions When a Parent Has a Mental Illness - Possibilities and Challenges.” *Practice* 33 (2): 103–18. https://doi.org/10.1080/09503153.2020.1760814.

Zanden, Rianne A.P. Van Der, Paula A.M. Speetjens, Karlijn S.E. Arntz, and Simone A. Onrust. 2010. “Online Group Course for Parents with Mental Illness: Development and Pilot Study.” *Journal of Medical Internet Research* 12 (5). https://doi.org/10.2196/jmir.1394.
